# Opportunities for personalised follow‐up care among patients with breast cancer: A scoping review to identify preference‐sensitive decisions

**DOI:** 10.1111/ecc.13092

**Published:** 2019-05-09

**Authors:** Kelly M. de Ligt, Laurentine S.E. van Egdom, Linetta B. Koppert, Sabine Siesling, Janine A. van Til

**Affiliations:** ^1^ Department of Research Netherlands Comprehensive Cancer Organisation (IKNL) Utrecht The Netherlands; ^2^ Department of Health Technology and Services Research, Technical Medical Centre University of Twente Enschede The Netherlands; ^3^ Department of Surgical Oncology Erasmus MC Cancer Institute, University Medical Centre Rotterdam Rotterdam The Netherlands

**Keywords:** breast neoplasms, follow‐up care, personalised health care, scoping review, shared decision‐making, survivorship

## Abstract

**Introduction:**

Current follow‐up arrangements for breast cancer do not optimally meet the needs of individual patients. We therefore reviewed the evidence on preferences and patient involvement in decisions about breast cancer follow‐up to explore the potential for personalised care.

**Methods:**

Studies published between 2008 and 2017 were extracted from MEDLINE, PsycINFO and EMBASE. We then identified decision categories related to content and form of follow‐up. Criteria for preference sensitiveness and patient involvement were compiled and applied to determine the extent to which decisions were sensitive to patient preferences and patients were involved.

**Results:**

Forty‐one studies were included in the full‐text analysis. Four decision categories were identified: “surveillance for recurrent/secondary breast cancer; consultations for physical and psychosocial effects; recurrence‐risk reduction by anti‐hormonal treatment; and improving quality of life after breast cancer.” There was little evidence that physicians treated decisions about anti‐hormonal treatment, menopausal symptoms, and follow‐up consultations as sensitive to patient preferences. Decisions about breast reconstruction were considered as very sensitive to patient preferences, and patients were usually involved.

**Conclusion:**

Patients are currently not involved in all decisions that affect them during follow‐up, indicating a need for improvements. Personalised follow‐up care could improve resource allocation and the value of care for patients.

## 1 BACKGROUND

Breast cancer is the most common form of cancer among women worldwide (Bray et al., 2018). The five‐year relative survival for early stage breast cancer is high, with rates exceeding 96% for stage I and 86% for stage II disease (Howlader et al., 2017; Janssen‐Heijnen et al., 2014). International guidelines state that the goals of breast cancer follow‐up care are to detect recurrent disease or new malignancies at an early stage, and to inform and counsel patients about the physical and psychosocial (late) effects of therapy (Grunfeld, Dhesy‐Thind, & Levine, 2005; IKNL, 2012; Runowicz et al., 2016; Senkus et al., 2015). Schemes for detecting recurrences often comprise annual physical and mammographic examinations for at least five years, depending on the patient's age, genetic predisposition and/or tumour characteristics. Consultations that seek to detect physical and psychosocial effects are often linked to the visits for recurrence detection and are most frequently planned during the first year of follow‐up (IKNL, 2012; Senkus et al., 2015).

At present, arrangements for follow‐up suboptimally meet the needs of patients with breast cancer, and there is concurrently a growing demand for personalised care planning within cancer follow‐up care (DH Macmillan Cancer Support & NHS Improvement, 2010; Donnelly, Hiller, Bathers, Bowden, & Coleman, 2007; Montgomery, Krupa, & Cooke, 2007; Zorginstituut Nederland, 2016). Such personalised follow‐up care could be based on the patient's individual risk of recurrence for the length and/or frequency of surveillance (IJzerman, Hans, Siesling, & Klaase, 2011; Witteveen et al., 2015), or on the type of treatment, and therefore, the management of treatment‐induced (late) effects and complaints (IKNL, 2012; Senkus et al., 2015). Moreover, cancer survivors might experience very different psychosocial consequences after the disease and treatment, including fear of recurrence, sleeping difficulties, cognitive issues, fatigue and sexual issues (Ewertz & Jensen, 2011). Each of these effects requires a personalised follow‐up strategy. Patient preferences about the preferred form and content of the follow‐up care have been reported in previous studies (Kimman, Dellaert, Boersma, Lambin, & Dirksen, 2010; Murchie et al., 2016).

Since the advent of value‐based health care, there have been ongoing efforts to improve care quality by adding value throughout an individual patient's journey from diagnosis, through treatment, and to follow‐up care (Porter & Teisberg, 2007). A way to meet this goal of personalised care is to include patients and their preferences in the decision‐making process. For example, in the shared decision‐making (SDM) process, decisions are based on both the best available (medical) evidence and the patients’ needs and values. Preference‐sensitive care involves making treatment decisions with significant trade‐offs that should reflect a patient's personal values and preferences. Besides, only when patients have enough information to make an informed choice, a decision can be made (Légaré, Ratté, Gravel, & Graham, 2008). This means that the quality of this SDM process might affect the eventual effect on the value of care, in terms of outcomes, costs and organisational effort (van de Haterd, Voogdt‐Pruis, Raats, van den Brink, & van Veenendaal, 2016).

In the present study, we hypothesised that decisions about breast cancer follow‐up are sensitive to patient preferences, and that it is an option to include SDM in the follow‐up care of these patients. Thus, we aimed to discover the potential for personalising follow‐up care among patients with breast cancer by exploring the evidence on preferences for, and patient involvement in, decisions about breast cancer follow‐up care.

## 2 METHODS

The review was registered in PROSPERO (reference No.: CRD42018082501) ([Link]).

### 2.1 Search strategy

Three research questions were posed: (a) “what decisions are made during follow‐up about content or form of follow‐up care for breast cancer survivors?” (b) “to what extent are these decisions sensitive to patient preferences?” and (c) “to what extent and how are patients with breast cancer involved in making these decisions?” The literature was searched separately for each question, between 18th July and 25th September 2017, in the MEDLINE (accessed through PubMed), PsycINFO (accessed through Ovid) and EMBASE databases (Table 1). We included any study that discussed decisions made or interventions applied during follow‐up for breast cancer, provided it was written in English and published in the last 10 years (2008–2017). The time restriction was set because breast cancer care and treatment have changed significantly over previous decades. The follow‐up period was defined as the time period after surgery for breast cancer.

**Table 1:** Search strategy per research question^a^ for MEDLINE (accessed through PubMed), PsycINFO (accessed through Ovid), and EMBASE

Search words	Databases			Research question^a^		
	MEDLINE (PubMed)	PsychINFO (Ovid)	EMBASE	*1*	*2*	*3*
Breast cancer	(("breast"[MeSH Terms] OR "breast"[All Fields]) AND ("neoplasms"[MeSH Terms] OR "neoplasms"[All Fields] OR "cancer"[All Fields])) OR ("neoplasms"[MeSH Terms] OR "neoplasms"[All Fields] OR "malignancy"[All Fields]) OR ("tumour"[All Fields] OR "neoplasms"[MeSH Terms] OR "neoplasms"[All Fields] OR "tumor"[All Fields]) OR ("carcinoma"[MeSH Terms] OR "carcinoma"[All Fields]) OR "neoplasms"[MeSH Terms] OR "neoplasms"[All Fields] OR "neoplasm"[All Fields] OR "mass"[All Fields] OR Nodule[All Fields] OR ("cysts"[MeSH Terms] OR "cysts"[All Fields] OR "cyst"[All Fields])	exp BREAST NEOPLASMS/ OR (exp BREAST/ AND exp NEOPLASMS/ ) OR breast cancer.mp OR ((breast.mp OR exp BREAST/ ) AND (cancer.mp OR neoplasm*.mp OR carcin*.mp OR tumor*.mp OR tumour*.mp OR metasta*.mp OR malig*.mp))	breast cancer'/exp OR (breast:ti,ab AND carcinoma*:ti,ab) OR (breast:ti,ab AND cancer*:ti,ab) OR (breast:ti,ab AND neoplasm*:ti,ab) OR (breast:ti,ab AND tumour*:ti,ab) OR (breast:ti,ab AND tumor*:ti,ab) OR (breast:ti,ab AND metasta*:ti,ab) OR (breast:ti,ab AND malig*:ti,ab) OR ('breast'/exp AND (neoplas*:ti,ab OR cancer*:ti,ab OR carcin*:ti,ab OR tumor*:ti,ab OR tumour*:ti,ab OR metasta*:ti,ab OR malig*:ti,ab OR 'neoplasm'/exp))	x	x	x
Follow‐up	follow‐up[All Fields] OR ("aftercare"[MeSH Terms] OR "aftercare"[All Fields] OR ("after"[All Fields] AND "treatment"[All Fields]) OR "after treatment"[All Fields]) OR "survival"[MeSH Terms] OR "survival"[All Fields] OR "survivorship"[All Fields] OR (care[All Fields] AND plan[All Fields]) OR care[All Fields] OR surveillance [All Fields]	follow‐up.mp. OR exp POSTTREATMENT FOLLOWUP/ OR followup.mp OR aftercare.mp OR after‐care.mp OR exp Aftercare/ OR ((exp PATIENTS/ or patient.mp) AND (monitoring.mp. or exp MONITORING/)) OR after treatment.mp OR exp Survivors/ OR survival.mp OR survivorship.mp OR exp Treatment Planning/ OR care plan.mp OR surveillance.mp	follow up':ti,ab OR 'aftercare':ti,ab OR 'aftercare'/de OR (after NEAR/1 treatment):ti,ab OR 'survival':ti,ab OR 'survival'/de OR 'survivorship'/de OR 'survivorship':ti,ab OR (care NEAR/1 plan):ti,ab OR 'surveillance'/de OR 'surveillance'	x		x
Decision‐making	("Decisions"[Journal] OR "decisions"[All Fields]) AND ("decision support techniques"[MeSH Terms] OR ("decision"[All Fields] AND "support"[All Fields] AND "techniques"[All Fields]) OR "decision support techniques"[All Fields] OR ("decision"[All Fields] AND "analysis"[All Fields]) OR "decision analysis"[All Fields])	decision‐making.mp. or exp Decision Making/ OR ((support techniques.mp) AND (decision.mp)) OR ((support.mp) AND (techniques.mp)) OR decision support techniques.mp OR ((decision.mp) AND (analysis.mp)) OR decision analysis.mp	decision making'/de OR 'decision making':ti,ab OR ('decision'/de OR decision AND ('support'/de OR support) AND techniques) OR 'decision'/de OR decision AND ('analysis'/de OR analysis)	x		
Preference‐sensitive decisions	preference[All Fields] AND sensitive[All Fields] AND ("Decisions"[Journal] OR "decisions"[All Fields])	preference‐sensitive.mp	preference sensitive':ti,ab		x	
Shared decision‐making	decision making[MeSH Terms] OR ("decision"[All Fields] AND "making"[All Fields]) OR "decision making"[All Fields] OR ("shared"[All Fields] AND "decision"[All Fields] AND "making"[All Fields]) OR "shared decision making"[All Fields]	((shared.mp) AND (decision‐making.mp or exp Decision Making/))	shared decision making'/de OR 'shared decision making'			x

(a) What are the common complaints and issues that can occur for woman treated for breast cancer with curative intent for which decisions have to be made with regard to management within five years after curative treatment? (b) To what extent are decisions with regard to the management of these complaints preference‐sensitive? (c) To what extent and how are patients with breast cancer involved in making these follow‐up‐related decisions?

After removing duplicates, study titles and abstracts were screened by two independent screeners (KdL and LE). Studies were excluded if they did not include patients with breast cancer, did not discuss follow‐up, did not describe actual decision‐making or did not describe the patients' roles in decision‐making. Studies were also excluded if they included patients receiving palliative treatment. Full texts were retrieved for the remaining studies. Those without full‐text articles were excluded after attempt to contact the corresponding authors to access the text. EndNote (Clarivate Analytics ) was used to manage all search results.

### 2.2 Quality assessment

The quality of the included studies was assessed by the Critical Appraisal Skills Programme checklist, comprising criteria for qualitative studies, randomised controlled trails, cohort studies and systematic reviews. Criteria could be scored with a positive or negative response; when criteria were not applicable or unknown/unable to be assessed, this was recorded as well ([Link]). First, we determined the study design for each included study, provided this was not already described in the study's method section. Studies were deemed of sufficient quality when half or more of the criteria could be scored positive, provided there was a clear aim or research question.

### 2.3 Analyses

First, we identified the decisions were made or could be made about content or form of follow‐up care delivered to breast cancer patients. Second, criteria were compiled to determine whether decisions were sensitive to patient preferences and whether patients were involved in making the decisions. Third, these criteria, in turn, were used to assess the degree to which decisions were sensitive to patient preferences and the extent to which patients were involved in making these decisions.

Criteria for preference sensitiveness (PS0‐5) were based on the definition by Van der Weijden et al. (2013). Decisions were considered preference‐sensitive if the following criteria were met:

0. There were multiple options available (PS0); *and*
1. Options had potential favourable and unfavourable outcomes, leading to an individual trade‐off (PS1); *or*
2. Options did not differ in terms of favourability of the outcomes, or (un)favourable outcomes were equally (un)desirable (PS2); *or*
3. There was insufficient evidence about favourable or unfavourable outcomes to determine the best option (PS3); *or*
4. The potential risks of an option were high, regardless the potential benefits of this option (PS4); *or*
5. The outcomes were highly dependent on patient cooperation, or the actions required for the preferred option had high impact on the patient's lifestyle (PS5).

Criteria for the extent of patient involvement (SDM1‐7) were based on the conditions set by Légaré et al. (2008) and the components described by Coulter and Collins (2011):

1. The decision was preference‐sensitive (SDM1); *and*
2. There was sufficient time to make a decision (SDM2); *and/or*
3. The patient was capable and sufficiently informed to make a decision (SDM3); *and/or*
4. There was a belief that SDM would lead to better patient outcomes (SDM4); *and/or*
5. The physician was motivated for SDM and clarified the options and preferences (SDM5); *and/or*
6. There was a belief that SDM will lead to better clinical outcomes (SDM6); *and/or*
7. There was a system for recording, communicating, and implementing the patient's preferences (SDM7).

## 3 RESULTS

**Figure 1:**
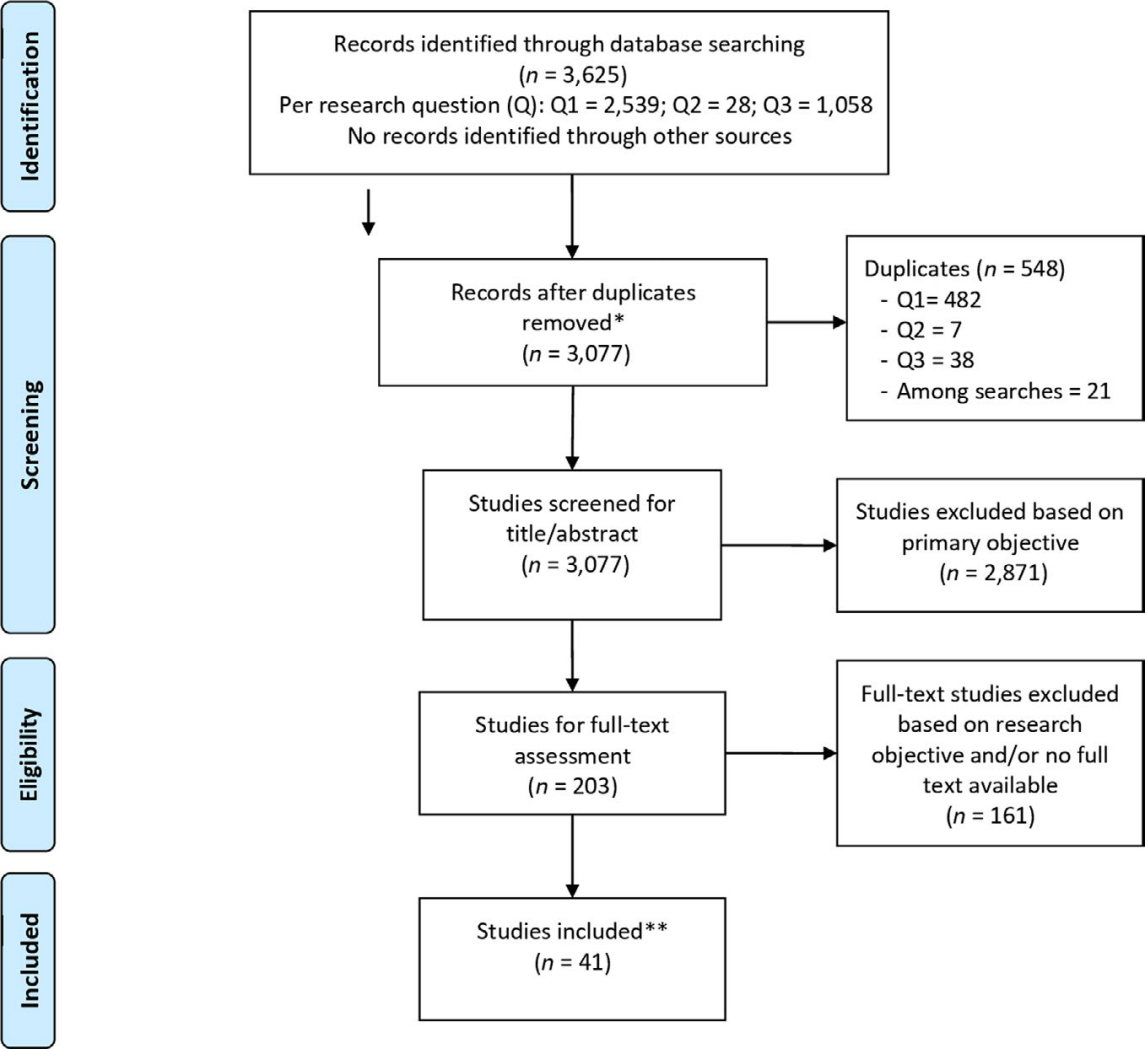
PRISMA flow chart of study inclusion. *Three literature searches were conducted (a search per research question), as shown in the identification box. Next, duplicates were removed from within each search, before being removed by cross‐checking between the searches. **One duplicate was removed from the studies that were finally included. From: Moher, Liberati, Tetzlaff, and Altman (2009). For more information, visit www.prisma-statement.org

Figure 1 summarises the selection process according to the PRISMA scheme. In total, 3,077 records were screened after removing duplicates (*n* = 2,539, 28, 1,058 per research question). After screening titles, abstracts and full texts, we finally included 41 studies.

Within the screened records, “*follow‐up*” often referred to the study design rather than the post‐treatment period, and “*preference‐sensitive*” was used little or infrequently, only appearing as a key word in 21 records. Studies also generally described gaps in patient involvement rather than care that was already well‐organised. Moreover, we excluded many studies (*n* = 2,871) that could not be related to the SDM criteria because they did not describe decision‐making about the content or form of follow‐up care. Another 11 studies were excluded because the full texts were not available. These were mainly studies published as conference abstracts, dissertations or books. Contact details were available for only five of the corresponding authors of these abstracts, and only one responded.

All included studies (*n* = 41) were rated as valuable in the quality assessment (Table S2). Most studies employed a design with surveys (*n* = 11) or interviews (*n* = 16); comprising focus groups, needs assessments and semi‐structured/directed/open‐ended interviews. The survey‐based studies included larger samples (*n* = 5–41), whereas the interview‐based studies included smaller groups (*n* = 5–41). Less common methods included studies of electronic health records (*n* = 1) and internet fora (*n* = 1). Randomised Controlled Trials (RCT) designs were used for studies about lifestyle interventions (*n* = 2) and SDM‐related tools about breast reconstruction (*n* = 3).

Table 2 summarises the preference‐sensitive aspects (criterion PS) and aspects of patient involvement (criterion SDM) for each decision about the content or form of follow‐up care. Decisions were classified into those concerning (a) “surveillance for recurrent or secondary breast cancer”; (b) “consultations for physical and psychosocial (late) effects”; (c) “recurrence‐risk reduction by anti‐hormonal treatment”; and (d) “improving quality of life after breast cancer.” Results are described in more detail below. Table S1 summarises the included studies.

**Table 2:** Preference sensitiveness and patient involvement, based on the pre‐specified criteria for each decision about the content or form of follow‐up care

Decision	Degree in which decisions are preference‐sensitive (criteria PS)	Conditions for shared decision‐making (criteria SDM)
Surveillance for recurrent or secondary breast cancer		
Form (Brandzel et al., 2017; Klaassen et al., 2017)	0) Women underwent various types of surveillance imaging (not specified), although almost all woman received mammographic examination. Some also received MRI (Brandzel et al., 2017). 1) Some women stated that they would prefer a false‐positive result with follow‐up procedures, other women wanted to avoid false‐positive results and follow‐up procedures because the additional tests caused too much worry, physical discomfort and potential expense. 1) Some women whose breast cancer was not found with screening mammography had less trust in mammography. Other women were confident in mammography and did not feel the need for reassurance from additional imaging modalities (Brandzel et al., 2017). 5) Cost and insurance coverage was an important topic that sometimes affected participant preferences (Brandzel et al., 2017).	3) Patients reported a need for information about the transition to surveillance care (Brandzel et al., 2017). Women reported feeling confusion about the choices for surveillance imaging or about the frequency of imaging examinations (Brandzel et al., 2017). 3) A point of improvement was women's understanding of (the goal of) surveillance (Brandzel et al., 2017). 5) Women reported trust in their providers and relied on providers for imaging decision‐making (Brandzel et al., 2017). Most participants reported that either their oncologist or surgeon recommended and made the referrals for their imaging type and frequency after treatment (Brandzel et al., 2017). 7) Although some patients received a detailed survivorship care plan, others reported that they did not receive clear information (Brandzel et al., 2017). 7) To promote SDM about form and frequency of follow‐up, Klaassen et al (Klaassen et al., 2017) suggest a follow‐up decision aid.
Frequency (Brandzel et al., 2017; Klaassen et al., 2017)	0) Many patients received surveillance mammography more often than the recommended annual frequency. 1) Most women were satisfied with the frequency or wanted more frequent surveillance to reassure they did not have a recurrent breast cancer (Klaassen et al., 2017). However, women also reported that breast imaging caused anxiety and was an uncomfortable experience (Brandzel et al., 2017).	3) A point of improvement was women's understanding of (the goal of) surveillance (Brandzel et al., 2017). 3) Women reported feeling confusion about the choices for surveillance imaging or about the frequency of imaging examinations (Brandzel et al., 2017). 5) Most of the participating patients had not discussed their preferences with any of the HPs, as they were afraid to damage the relationship they had with their HP (Klaassen et al., 2017). Most participants reported that either their oncologist or surgeon recommended and made the referrals for their imaging type and frequency after treatment (Brandzel et al., 2017). 7) To promote SDM about form and frequency of follow‐up, Klaassen et al (Klaassen et al., 2017) suggest a follow‐up decision aid.
Length	No studies identified	No studies identified
Hereditary testing (Rini et al., 2009)	0) Hereditary testing leads to information about the risk of secondary breast cancer or breast cancer in family members, affecting surveillance schemes or decisions about preventative options, such as contralateral prophylactic mastectomy 3) Inconclusive evidence: hereditary tests cannot always rule out completely the presence of genetic mutations. Counsellors typically provide these women with a qualitative estimate of their residual risk of carrying a mutation and of developing a second cancer. These risk estimates, which are based on various characteristics of a woman's family pedigree, are highly heterogeneous and entail a great deal of uncertainty. It is not currently clear how receiving an uninformative BRCA1/2 test result influences the difficulty of women's risk management decisions (Rini et al., 2009).	3) Because of the inconclusiveness of the results, decisions about risk reduction options can be underinformed (Rini et al., 2009). 5) The findings suggest that a substantial number of these women may benefit from assistance with risk management decision‐making. Genetic counsellors are one potential source of such assistance (Rini et al., 2009). 7) The development of a decision aid for women who receive uninformative BRCA1/2 test results may be warranted, particularly in light of the increasing availability and use of these tests (Rini et al., 2009).
Follow‐up consultations for physical and psychosocial (late) effects		
Form (Hudson et al., 2012; Klaassen et al., 2017)	1) Approximately, one quarter (24%) of participants reported seeking care from multiple providers, including a primary care physician (PCP, i.e., family physician, general internist, or gynaecologist) (Hudson et al., 2012). 1) Patients preferred consultations by a breast cancer specialist, possibly alternated with consultations with a nurse (Klaassen et al., 2017).	3) Women in their study reported that most patients were not offered options regarding structure and frequency of the aftercare appointments (Klaassen et al., 2017), although the option of between‐appointment calls with the nurse practitioner was provided to some patients (Klaassen et al., 2017). 5) Patients reported difficulty in expressing their need for options to their health professional (Klaassen et al., 2017). HPs felt that most patients want the same thing concerning aftercare (Klaassen et al., 2017). 7) To promote SDM about form and frequency of follow‐up, Klaassen et al (Klaassen et al., 2017) suggest a follow‐up decision aid.
Frequency (Hudson et al., 2012; Klaassen et al., 2017)	1) In all focus groups, patients mentioned that they would like either more personal attention from the HP, a higher frequency of physical checks‐ups to detect recurrences or more aftercare consultations in general (Klaassen et al., 2017).	3) Women in their study reported that most patients were not offered options regarding structure and frequency of the aftercare appointments (Klaassen et al., 2017), although the option of between‐appointment calls with the nurse practitioner was provided to some patients (Klaassen et al., 2017). 5) Physicians said that SDM is common practice in their healthcare facilities and in their own work as well and believed SDM made the patients feel positively involved in follow‐up related decisions. 5) Patients reported difficulty in expressing their need for options to their health professional (Klaassen et al., 2017). HPs felt that most patients want the same thing concerning aftercare (Klaassen et al., 2017). 7) To promote SDM about form and frequency of follow‐up, Klaassen et al. (2017) suggest a follow‐up decision aid. 7) However, not every patient is sufficiently activated and skilled to retrieve the care they require [29].
Length (Hudson et al., 2012; Klaassen et al., 2017)	0) All participants reported having received follow‐up care from a cancer specialist (i.e., medical/surgical/radiation oncologist) within the past year (Hudson et al., 2012).	
Recurrence‐risk reduction by anti‐hormonal treatment		
Treatment with adjuvant anti‐hormonal therapy: initiation (Bluethmann et al., 2017; Neugut et al., 2012).	0) Therapy initiation (Bluethmann et al., 2017; Neugut et al., 2012). 0) 96% of patients were steered towards undergoing anti‐hormonal therapy, irrespective of expected benefits (Engelhardt et al., 2016). 1) Preliminary evidence suggests that prioritising fertility, along with concerns about side effects, leads to ET non‐initiation and early discontinuation (Benedict et al., 2017).	2) Patients might feel overwhelmed: decision is directly posed after surgery, while patients might still be processing this surgery (Engelhardt et al., 2016). 3) Educational materials about family‐building after cancer are still not consistently available or provided (Benedict et al., 2017). 5) Patients did not always get to make a decision or were steered towards the option favoured by the clinician (Engelhardt et al., 2016). 6) Non‐initiation was less likely in those who found the quality of patient/physician communication to be higher (Neugut et al., 2012).
Treatment with adjuvant anti‐hormonal therapy: adherence (Benedict et al., 2017; Bluethmann et al., 2017; Brauer et al., 2016; Cahir et al., 2015; Engelhardt et al., 2016; Hershman et al., 2016).	0) Therapy adherence (Benedict et al., 2017; Bluethmann et al., 2017; Brauer et al., 2016; Cahir et al., 2015; Engelhardt et al., 2016; Hershman et al., 2016). 1) Key enablers for adherent/persistent women were identified within the domain beliefs about consequences (breast cancer recurrence), intentions and goals (high‐priority), beliefs about capabilities (side effects) and behaviour regulation (managing medication; Cahir et al., 2015). Quality of life and attitudes towards ET at baseline were associated with non‐persistence (Hershman et al., 2016). Preliminary evidence suggests that prioritising fertility, along with concerns about side effects, leads to ET non‐initiation and early discontinuation (Benedict et al., 2017). 3) The adverse effects of AIs were difficult to disentangle from what women attributed to comorbid conditions or getting older. This challenge in attribution, coupled with less frequent contact with their oncology team, resulted in many women “winging it” or persisting with the AI despite significant struggles (Brauer et al., 2016). 4) Risk‐versus‐benefit trade‐off (Bluethmann et al., 2017; Brauer et al., 2016; Engelhardt et al., 2016): anti‐hormonal therapy is an established risk‐reduction strategy for recurrences and contralateral breast cancer versus severity of side effects (Benedict et al., 2017; Bluethmann et al., 2017; Brauer et al., 2016; Hershman et al., 2016). 5) Its effectivity is highly dependent of the patients cooperation (therapy adherence) (Cahir et al., 2015); highly affect the patient's lifestyle by its side effects (Bluethmann et al., 2017; Brauer et al., 2016).	3) Gaps in information provision (Bluethmann et al., 2017; Brauer et al., 2016; Cahir et al., 2015; Engelhardt et al., 2016), for instance about expected side effects or possible management strategies (Bluethmann et al., 2017). 5) Regarding persistence, many reported lack of professional guidance or support with respect to persisting with the AI, especially when adverse effects were present, and relied on a variety of self‐management strategies to maintain treatment with the AI (Brauer et al., 2016).
Menopausal symptoms following from breast cancer therapies (Balneaves et al., 2016; Sayakhot et al., 2012).	0) Identification and treatment of menopausal symptoms. 2) As there are limited other conventional treatment options available, patients reside in alternative treatments as mind‐body therapies and natural health products (Balneaves et al., 2016). 3) There is a lack of reliable and unambiguous information about these options (Sayakhot et al., 2012). 4) The potential risks of hormone replacement therapy, which is the customary and most effective treatment option, could be high. This option is usually avoided for breast cancer patients as it increases recurrence risks (Balneaves et al., 2016; Sayakhot et al., 2012).	3) Although 80% of women were given breast cancer information, only 54% were given menopause information at diagnosis. Women were least satisfied (26%) with information regarding the long‐term complications of menopause (Sayakhot et al., 2012). 3) A lack of reliable and unambiguous information about treatment options for menopausal symptoms was reported (Balneaves et al., 2016; Sayakhot et al., 2012). 3) Some women were not aware their symptoms were menopause, induced by their cancer treatment— and not a temporary, remediable effect. Although many of the women were informed that their menstrual cycles would end following treatment, they did not fully realise the implications and meaning of the associated physiological changes. The women were surprised by the sudden onset and intensity of their menopausal symptoms (Balneaves et al., 2016). 3) In addition to being inundated by the large volume of information, the women were frustrated by the lack of conclusive information, particularly regarding complementary therapies. The majority of women were also frustrated by their inability to differentiate between credible and non‐credible information sources (Balneaves et al., 2016). 7) Balneaves et al. (2016), suggest a tool that summarises evidence for each option of menopausal treatment.
Improving quality of life		
Breast reconstruction (Alderman et al., 2011; Causarano et al., 2015; Fasse et al., 2017; Flitcroft et al., 2016; Fu et al., 2017; Hamnett & Subramanian, 2016; Heller et al., 2008; Lee et al., 2010; Morrow et al., 2014; Ogrodnik et al., 2016; Potter et al., 2013; Sherman et al., 2016; Temple‐Oberle et al., 2014; Zielinski et al., 2015)	0) Patients might decide to undergo breast reconstruction for years after surgery has taken place (Alderman et al., 2011; Sherman et al., 2016). 1) One‐third of mastectomy‐treated patients choose delayed reconstruction as they focussed on more on other treatment modalities (Alderman et al., 2011; Flitcroft et al., 2016). Two‐thirds of patients without reconstruction said this procedure was of no importance to them (Alderman et al., 2011); other reasons were that it was were “unnecessary” and “being practical” (Flitcroft et al., 2016), poor timing (25%), indecision (17%), desired method of reconstruction not available at treating facility (10%), persistent obesity (8.3%), continued smoking (4%), and reason not specified (35%) (Ogrodnik et al., 2016), it is not essential for their mental state, or they fully accepted their appearance after mastectomy (Zielinski et al., 2015). Older patients (>60 years) were less likely to choose for breast reconstruction (Flitcroft et al., 2016). Patients spoke about breasts as a function of their roles as a wife or mother, eliminating the need for breasts when these roles were fulfilled (Fu et al., 2017). Many addressed the fear of multiple operations (Fu et al., 2017; Zielinski et al., 2015). 4) A breast reconstruction is a major and invasive surgery (Alderman et al., 2011; Causarano et al., 2015; Flitcroft et al., 2016; Fu et al., 2017; Hamnett & Subramanian, 2016), regardless of the vast part of included studies that recognised the positive psychosocial effects that BR yields (Causarano et al., 2015; Flitcroft et al., 2016; Potter et al., 2013; Zielinski et al., 2015) and the importance of breast reconstruction for mastectomy patients (Alderman et al., 2011; Fasse et al., 2017; Fu et al., 2017). Half of the respondents was concerned about surgical complications and interference with cancer surveillance (Alderman et al., 2011), or post‐mastectomy radiotherapy might interfere with reconstruction (Flitcroft et al., 2016).	1) Within several studies, the preference‐sensitive nature of breast reconstruction decisions was literally appointed (Causarano et al., 2015; Lee et al., 2010; Ogrodnik et al., 2016). 3) Information provision could be improved (Alderman et al., 2011; Causarano et al., 2015; Fu et al., 2017; Hamnett & Subramanian, 2016; Heller et al., 2008; Morrow et al., 2014; Ogrodnik et al., 2016; Potter et al., 2013). The older patient is less likely to do research independently (Hamnett & Subramanian, 2016). 4) SDM about breast reconstruction yields positive effects as lower decisional conflict and higher satisfaction with information (Sherman et al., 2016). 5) Patients felt involved in the decision‐making process (Kadmon et al., 2016; Morrow et al., 2014). 7) Already several decision aids were developed for breast reconstruction (Causarano et al., 2015; Heller et al., 2008; Sherman et al., 2016; Temple‐Oberle et al., 2014).
Breast reconstruction techniques (Causarano *N* et al., 2015, Potter et al., 2013, Sherman et al., 2016, Temple‐Oberle et al., 2014).	0) In the decision to undergo a BR, there are multiple options of autologous or implant‐based BR, each leading to its own outcomes (criterion PS1) (Causarano et al., 2015; Potter et al., 2013; Sherman et al., 2016; Temple‐Oberle et al., 2014). 1) Patients placed greater importance on avoiding use of a prosthesis (Lee et al., 2010).	
Getting pregnant after breast cancer (Benedict et al., 2017; Corney & Swinglehurst, 2014; Gorman et al., 2011; Hsieh & Huang, 2017).	0) Getting pregnant after cancer treatment. 1) A wide variety in level of concern about fertility was noted, as this depends on personal circumstances, values and expectations (Gorman et al., 2011; Hsieh & Huang, 2017). Management of fertility issues was heavily influenced by social and cultural perceptions about having children (Hsieh & Huang, 2017). 3) More than half of the participants (*n* = 9, 56%) were concerned about passing cancer‐positive genes to their child; they worried that cancer‐related treatment could affect the child's health in the future (Hsieh & Huang, 2017). 4) Women in the study proactively collected information about cancer, cancer treatment and pregnancy. They then weighed the personal risk–benefit between conceiving and contraception based on their assessment of their personal situation and condition. Patients worried whether breast cancer and the treatment had a negative effect on their child	3) Patients were not sufficiently informed about risks of getting pregnant (Corney & Swinglehurst, 2014). 3) All included studies stated that patient information about management of fertility could be improved (Corney & Swinglehurst, 2014; Gorman et al., 2011; Hsieh & Huang, 2017). 3) The study by Balneaves et al. (2017) about menopausal symptoms described that oncology providers stated that they felt ill‐equipped to inform patient about fertility issues management. 5) Participants reported having very good relationships with their oncologists, describing them as a trusted and valuable source of information when making critical treatment decisions. However, the relationship later became strained for some women who felt that their decisions about pregnancy were not supported (Gorman et al., 2011).
Pregnancy and anti‐hormonal treatment (Benedict et al., 2017; Corney & Swinglehurst, 2014; Gorman et al., 2011; Hsieh & Huang, 2017).	1) An important cause of non‐initiation of anti‐hormonal therapy is the prioritising of family‐building over the benefits of anti‐hormonal therapy (Benedict et al., 2017). 1) The patients increasing age during anti‐hormonal treatment administration may give a decline in fertility as well (Corney & Swinglehurst, 2014)	3) All included studies stated that patient information about management of fertility could be improved (Corney & Swinglehurst, 2014; Gorman et al., 2011; Hsieh & Huang, 2017). 3) The study by Balneaves et al. (2016) about menopausal symptoms described that oncology providers stated that they felt ill‐equipped to inform patient about fertility issues management. 3) Clinical efforts to improve adherence to endocrine therapy might need to consider patients’ family‐building goals during the course of treatment and to appropriately counsel patients according to their priorities and family‐building intentions. Educational materials about family‐building after cancer are still not consistently available or provided (Benedict et al., 2017).
Pre‐treatment artificial reproductive techniques (Corney & Swinglehurst, 2014; Zielinski et al., 2015)	0) Women choose from a range of options including ovarian stimulation, or oocyte or embryo cryopreservation (Corney & Swinglehurst, 2014). 1) Women without a partner that did not want to opt for the less successful oocyte preservation, had to find a donor to enable embryo cryopreservation (Zielinski et al., 2015). 1) All the women indicated that they would not use the embryos or oocytes if they were able to conceive naturally. However, this led to the moral dilemma on what they would do with the eggs or embryos (Corney & Swinglehurst, 2014).	2) Decisions had to be made quickly [37]; women felt they were informed too late about their options [38]. 5) No woman was offered supportive counselling to aid the decision pursuing artificial reproductive techniques (Corney & Swinglehurst, 2014).
Lifestyle changes (Carter et al., 2010; Shtaynberger & Krebs, 2016)		
Lifestyle changes (Carter et al., 2010; Shtaynberger & Krebs, 2016)	1) The trade‐off is aimed at weighing pros and cons of making a change, so‐called decisional balance (Shtaynberger & Krebs, 2016). Participants’ reasons for selecting a particular physical activity program are diverse. A variety of activity programmes might be necessary to fit the needs of cancer survivors (Carter et al., 2010). 5) The effect of lifestyle interventions is highly dependent of the patients cooperation.	
Alternative medicine (Holmes, Bishop, & Calman, 2017)		
Use of alternative and complemen‐tary medicine (Holmes et al., 2017)	3) Holmes et al. (2017) describe patients opting for complementary alternative medicine in general and whether and how they were supported in this decisions. information available on the internet plays a factor in the decision‐making process to use CAM, as it may be seen as the only comprehensive way to get information on CAM.	3) Many participants expressed a need for information after their cancer diagnosis and viewed the internet as the only accessible way to get information. Due to the unrestricted nature of the internet, many had concerns about the legitimacy of website content. 5) Patients mainly used the internet to inform themselves about this topic, as they experienced a lack of approval from their social network and healthcare providers (Holmes et al., 2017).

Abbreviations: AI: aromatase inhibitors; BR: breast reconstruction; CI: confidence interval; CAM: complementary alternative therapy; ET: endocrine therapy; HP: healthcare practitioner; IBR: immediate breast reconstruction; NBR: no breast reconstruction; RCT: randomised controlled trial.

Aspects of preference‐sensitive decisions (PS):

PS0) there are multiple options available.

PS1) options have various benefits in terms of (un)attractiveness that lead to an individual trade‐off.

PS2) options do not differ in terms of favourable and unfavourable outcomes, or (un)favourable outcomes are equally (un)desirable.

PS3) there is insufficient evidence on favourable and unfavourable outcomes to determine the best option.

PS4) potential risks of a certain option are high, regardless of the benefits of this option.

PS5) the outcomes are highly dependent on the patients cooperation with the required actions, the required actions for the best option (which can in the guidelines) has high impact on the patient's lifestyle.

Conditions for shared decision‐making (SDM):

SDM1) the decision is preference‐sensitive.

SDM2) there is sufficient time to make a decision.

SDM3) patient is capable and informed enough to make a decision.

SDM4) there is a belief that SDM will lead to better patient outcomes.

SDM5) physician is motivated for SDM and counsels decision support to clarify options and preferences.

SDM6) there is a belief that SDM will lead to better clinical outcomes.

SDM7) there is a system for recording, communication, and implementing the patients preferences.

### 3.1 Surveillance for recurrent or secondary breast cancer

Follow‐up aims to detect recurrent disease or new associated malignancies at an early stage through surveillance imaging (mammography and/or MRI) and physical examination (Grunfeld et al., 2005; IKNL, 2012; Runowicz et al., 2016; Senkus et al., 2015). Two included studies discussed decisions about the *form* and *frequency of surveillance imaging* (PS0) (Brandzel et al., 2017; Klaassen, Dirksen, Boersma, & Hoving, 2017). Klaassen et al. assessed the needs of Dutch patients and physicians with regard to an aftercare decision aid. Brandzel et al. then described the experiences and preferences for breast imaging among breast cancer survivors in the United States. The main *form* of surveillance tended to be mammography, though some also received MRI; however, the authors did not specify who received what type of surveillance imaging or the reasons for the differences. If their breast cancer initially was missed on mammography, patients sometimes lost trust in this method and preferred other imaging modalities. Furthermore, many patients received surveillance mammography more often than the recommended annual frequency without clinical indication (Brandzel et al., 2017). Patients preferred this higher *frequency* because it reassured them about the absence of recurrences (Brandzel et al., 2017; Klaassen et al., 2017). However, breast imaging also caused anxiety and was considered uncomfortable for many patients (Brandzel et al., 2017), suggesting scope for a trade‐off between burdens and benefits of surveillance imaging (PS1). Surveillance preferences were also affected by financial costs and insurance coverage (Brandzel et al., 2017), and therefore, the patient's willingness to bear these costs (PS5).

Little evidence was found for patient involvement in surveillance‐related decisions. Brandzel et al. found that physicians typically determined the imaging type and frequency of surveillance (SDM5), despite the opposing preferences and trade‐offs expressed by patients. The patient's understanding of the goal of surveillance could be improved here: patients felt confused about the options for the type of surveillance imaging and frequency of surveillance imaging, and expressed a need for information about the transition from treatment to surveillance care (SDM3). The aftercare decision aid produced by Klaassen et al. provides an overview of follow‐up options (SDM7) and could reduce information needs before initiating follow‐up. Surveillance *length* was not discussed in the literature.

*Hereditary testing* is most often performed during breast cancer diagnosis and may be less relevant during follow‐up (IKNL, 2012). However, Rini et al. (2009) described hereditary testing in women with a history of breast cancer. Hereditary testing leads to information about the risk of secondary breast cancer and/or risk of breast cancer or ovarian cancer in family members. This can affect surveillance schemes or preventative options, such as contralateral prophylactic mastectomy (PS0).

### 3.2 Consultations for physical and psychosocial (late) effects

The second goal of follow‐up is informing and counselling patients about the physical and psychosocial (late) effects of treatment (Grunfeld et al., 2005; IKNL, 2012; Runowicz et al., 2016; Senkus et al., 2015). Two studies described decision‐making regarding the *form, frequency and length of follow‐up consultations* within follow‐up care (PS0). Patients preferred more personal attention from their physician and a higher *frequency* of oncology‐led aftercare than was offered (current situation not defined), which gave them more security about their health (Klaassen et al., 2017). Regarding the *length* of follow‐up consultation, all USA‐based participants in a study by Hudson et al. had received follow‐up care from a cancer specialist within the previous year, even though the time since their last active cancer treatment ranged from three to seventeen years; however, decisions about length were not discussed further (Hudson et al., 2012). Regarding the *form* of consultations, patients preferred consultations by a breast cancer specialist, possibly alternated with nurse consultations (PS1) (Klaassen et al., 2017). Regardless of these preferences, patients were rarely offered options about the frequency, form or length of consultations, indicating low patient involvement.

By contrast, most physicians stated that SDM was common practice in their healthcare facilities and in their own work, and reported that using SDM made the patients feel positively involved in decisions related to follow‐up (SDM5) (Klaassen et al., 2017). Referral to other medical specialists or care providers during follow‐up was not specifically described. However, 24% of patients sought care from multiple providers, including a primary care provider, general internist or gynaecologist (Hudson et al., 2012).

### 3.3 Recurrence‐risk reduction by anti‐hormonal treatment

Seven studies described *treatment decisions about anti‐hormonal therapy* (Benedict, Thom, Teplinsky, Carleton, & Kelvin, 2017; Bluethmann et al., 2017; Brauer, Ganz, & Pieters, 2016; Cahir et al., 2015; Engelhardt et al., 2016; Hershman et al., 2016; Neugut et al., 2012). This consisted of tamoxifen or aromatase inhibitor use to increase locoregional tumour control and survival, given for a minimum of five consecutive years, and continuing during follow‐up (IKNL, 2012). Respectively, there were two and five studies on decisions regarding *therapy initiation* (Bluethmann et al., 2017; Neugut et al., 2012) and *therapy adherence* (Benedict et al., 2017; Bluethmann et al., 2017; Brauer et al., 2016; Cahir et al., 2015; Hershman et al., 2016). Within the literature, therapy initiation was rarely regarded as a preference‐sensitive decision: one study described that 96% of patients were steered towards anti‐hormonal therapy, irrespective of the expected benefit (Engelhardt et al., 2016); in another study, patients felt obliged to take the therapy (PS0) (Bluethmann et al., 2017). However, the decision about *anti‐hormonal therapy* is not an one‐off decision: four studies described that the decision to adhere to anti‐hormonal therapy leads to patients making an ongoing risk‐versus‐benefit trade‐off between the risk‐reducing effect of treatment and the severity of treatment‐induced side effects (PS4) (Benedict et al., 2017; Bluethmann et al., 2017; Brauer et al., 2016; Cahir et al., 2015; Hershman et al., 2016). Non‐adherent patients in two studies felt unable to cope with side effects that severely affected their lives (PS5) (Bluethmann et al., 2017; Brauer et al., 2016). Three studies reported that professional guidance or support from physicians for managing these side effects could be improved (Benedict et al., 2017; Brauer et al., 2016; Cahir et al., 2015). Such guidance is important, because patients can better persevere with side effects if they have a high belief in their ability to manage and control their medication and side effects (PS1) (Cahir et al., 2015). However, four studies reported gaps in providing information about expected side effects (Benedict et al., 2017; Bluethmann et al., 2017; Engelhardt et al., 2016) or their management (SDM3) (Bluethmann et al., 2017; Brauer et al., 2016).

Frequently reported effects of anti‐hormonal therapy were menopausal symptoms and joint pain, with cognitive decline and cardiac distress also occurring, but less frequently (Bluethmann et al., 2017). Two studies specifically discussed the identification and treatment of *treatment‐induced menopausal symptoms* (PS0) (Balneaves et al., 2016; Sayakhot, Vincent, & Teede, 2012), such as hot flashes, weight gain, loss of sexuality and increased osteoporosis. Symptom treatment was considered a preference‐sensitive decision because hormone replacement therapy is the customary and most effective option, even though it increases the risk of recurrence and should be avoided in patients with breast cancer (PS4) (Balneaves et al., 2016; Sayakhot et al., 2012). However, there are few alternatives (PS2), with these limited to various lifestyle changes, pharmaceutical options and complementary treatments (e.g., mind‐body therapies and natural health products) (Balneaves et al., 2016). As both studies reported, a lack of reliable and unambiguous information about these options makes it difficult to select the best option (PS3). Concerning this dilemma, patients were frustrated by the lack of conclusive information, particularly about complementary therapies, and by an inability to differentiate between credible and non‐credible information sources (SDM3). Balneaves et al. suggested using an SDM‐tool that could summarise credible information about accepted options and thus facilitate decision‐making (SDM7). Two‐third of patients in this study still used complementary therapy to manage symptoms, despite the lack of information (Balneaves et al., 2016).

### 3.4 Improving quality of life after breast cancer treatment

This topic was subdivided into three subtopics. Sixteen studies focused on *delayed breast reconstruction*, two on *lifestyle changes*, and four on *getting pregnant after breast cancer*.

Breast reconstruction yields positive psychosocial effects (Causarano et al.., 2015; Flitcroft et al., 2016; Potter, Mills, Cawthorn, Wilson, & Blazeby, 2013; Zielinski, Lorenc‐Podgorska, & Antoszewski, 2015) and may contribute to the patients well‐being after breast cancer. Although some, if not most decisions about breast reconstruction are made before surgical treatment, resulting in immediate breast reconstruction, some patients and/or clinicians delay the decision about breast reconstruction until after treatment. Patients must then first decide whether to undergo delayed breast reconstruction, and when they do, decide which reconstruction technique should be used (PS0). Decisions about delayed breast reconstruction can remain relevant years after tumour surgery (Alderman et al., 2011; Sherman et al., 2016) and have been recognised as highly preference‐sensitive in three studies (Causarano et al., 2015; Lee, Hultman, & Sepucha, 2010; Ogrodnik, Maclennan, Weaver, & James, 2016). Furthermore, seven studies indicated that breast reconstruction yields positive psychosocial effects (Causarano et al., 2015; Flitcroft et al., 2016; Potter et al., 2013; Zielinski et al., 2015) and that it is an important option for patients who have undergone mastectomy (Alderman et al., 2011; Fasse et al., 2017; Fu, Chang, Chen, & Rohde, 2017). In three studies, common reasons for opting to delay breast reconstruction rather than undergoing immediate breast reconstruction were reported, and it was concluded that either patients wanted to focus on other treatment modalities first (Alderman et al., 2011; Flitcroft et al., 2016), or that the desired technique was not available at their facility (Ogrodnik et al., 2016). Patients generally refused breast reconstruction if they felt it was not important, urgent (Alderman et al., 2011), or necessary, or feared undergoing further surgery (Flitcroft et al., 2016). Thus, apart from medical contraindications, decisions about undergoing breast reconstruction were affected by its timing and individual decisions about trade‐offs (PS1). Regardless of the potential for positive psychosocial effects (Causarano et al., 2015; Flitcroft et al., 2016; Potter et al., 2013; Zielinski et al., 2015), risks of breast reconstruction can be high (PS4). Indeed, it is a major and invasive surgery (Alderman et al., 2011; Causarano et al., 2015; Flitcroft et al., 2016; Fu et al., 2017; Hamnett & Subramanian, 2016), and patients have reported concerns about surgical complications, and interference with cancer surveillance (Alderman et al., 2011), or post‐mastectomy radiotherapy (Flitcroft et al., 2016). There are also multiple options, such as autologous or implant‐based breast reconstruction (PS0), with each associated with different outcomes (PS1) (Causarano et al., 2015; Potter et al., 2013; Sherman et al., 2016; Temple‐Oberle et al., 2014).

Current patient involvement in decisions about breast reconstruction appeared to be high: fifteen studies described elements of patient involvement or SDM (Alderman et al., 2011; Causarano et al., 2015; Fasse et al., 2017; Flitcroft et al., 2016; Fu et al., 2017; Hamnett & Subramanian, 2016; Heller, Parker, Youssef, & Miller, 2008; Kadmon, Noy, Billig, & Tzur, 2016; Lee et al., 2010; Morrow et al., 2014; Ogrodnik et al., 2016; Potter et al., 2013; Sherman et al., 2016; Zielinski et al., 2015), and patients in two studies specifically reported feeling involved in decision‐making (SDM5) (Kadmon et al., 2016; Morrow et al., 2014). SDM about breast reconstruction led to less conflict around decisions and to more satisfaction with the information provided (SDM4) (Sherman et al., 2016). By contrast, four studies reported that patients experienced decision‐making uncertainty (Alderman et al., 2011; Fu et al., 2017; Sherman et al., 2016; Zielinski et al., 2015) and eight studies recommended further improvement of information provision (SDM3) (Alderman et al., 2011; Causarano et al., 2015; Fu et al., 2017; Hamnett & Subramanian, 2016; Heller et al., 2008; Morrow et al., 2014; Ogrodnik et al., 2016; Potter et al., 2013). This could be addressed by using one of four decision aids that have been developed (SDM7) (Causarano et al., 2015; Heller et al., 2008; Sherman et al., 2016; Temple‐Oberle et al., 2014).

In younger patients, breast cancer treatment can interfere with the desire to have a family. Four studies described the decision to *get pregnant after treatment for breast cancer* (Benedict et al., 2017; Corney & Swinglehurst, 2014; Gorman, Usita, Madlensky, & Pierce, 2011; Hsieh & Huang, 2017). Although this decision may feel like a risk, there is consensus that pregnancy following breast cancer is safe (Corney & Swinglehurst, 2014). Nevertheless, both patients and physicians have expressed concerns about the potential for pregnancy to increase recurrence risk in patients with hormone‐sensitive breast cancer (PS4) (Corney & Swinglehurst, 2014; Gorman et al., 2011; Hsieh & Huang, 2017). Patients not only felt under informed (SDM3) (Corney & Swinglehurst, 2014), but also, patients worried whether breast cancer and its treatment would negatively affect the health of a future child (PS4) (Corney & Swinglehurst, 2014; Hsieh & Huang, 2017). In general, there was a wide variety in the level of concern about fertility and getting pregnant. The importance of family‐building depended on personal circumstances, values and expectations (Corney & Swinglehurst, 2014; Gorman et al., 2011; Hsieh & Huang, 2017). In a study of Chinese breast cancer survivors, social and cultural perceptions about having children were important motives (PS1) (Hsieh & Huang, 2017). Although all three included studies described patient involvement in decisions about fertility management, it was also noted that the information provided could be improved (SDM3).

Anti‐hormonal therapy may cause infertility in pre‐menopausal patients. Those on anti‐hormonal therapy may therefore have to wait to the end of the treatment period (i.e., 5 years), while may be accompanied by an age‐related decline in fertility (PS1). In some patients, oncologists were willing to discuss the option of a reduced duration of anti‐hormonal treatment (Corney & Swinglehurst, 2014). Another study recognised the need to counsel patients about family‐building periodically during anti‐hormonal treatment (Benedict et al., 2017). Indeed, fertility counselling may remain important throughout follow‐up because treatment‐affected fertility may have negative psychosocial consequences (Gorman et al., 2011; Hsieh & Huang, 2017).

Chemotherapy treatment can also lead to reduced fertility. Therefore, patients should have the option to choose from a range of artificial reproductive techniques, including ovarian stimulation, and oocyte or embryo cryopreservation, before treatment (PS0) (Corney & Swinglehurst, 2014). These decisions will also affect decision‐making during follow‐up, for instance, patients who have opted for artificial reproductive techniques before treatment will have to decide on what to do with their preserved oocytes or embryos after treatment (PS0). All patients in a study by Corney and Swinglehurst (2014) indicated that they would not use the embryos or oocytes if they were able to conceive naturally, leading to moral decision about what to do with the oocytes or embryos.

Quality‐of‐life improvements after cancer may be found by implementing *lifestyle changes*. Two RCTs described a lifestyle intervention and the reasons why patients did and did not participate (PS0) (Carter et al., 2010; Shtaynberger & Krebs, 2016). Shtaynberger and Krebs (2016) described how decisions about physical activities and fruit and vegetable intake were based on an individual weighing the pros and cons of making a change (the so‐called decisional balance) (PS1). Carter et al. (2010) described the reasons for cancer patients to participate in either of two physical activity programmes (walking or “dragon boat” rowing) offered in their RCT. They reported that decisions were based on physical (health benefits), social (meeting new people, learning new skills) and practical (time investment, scheduling) considerations, but did not state whether the decision was discussed with a physician.

## 4 DISCUSSION

In this study, we aimed to assess the potential to personalise follow‐up care for patients after breast cancer treatment, by exploring the evidence on patient preferences for, and patient involvement in decisions about follow‐up care. We identified many decisions that needed to be made during follow‐up, including those related to surveillance imaging, follow‐up consultations, anti‐hormonal treatment, treatment‐induced menopausal symptoms and lifestyle changes. Moreover, we identified decisions that were made during treatment, but that required additional decisions during follow‐up, such as delayed breast reconstruction, hereditary testing and pregnancy. The literature revealed that there was a large variety in the degree of preference sensitiveness and patient involvement with each decision during follow‐up. Decisions about delayed breast reconstruction, for instance, were among those shown to be highly preference‐sensitive and for which many indications for patient involvement existed. Equally, however, decisions were identified for which patients exhibited preferences, but for which they were not necessarily involved. Notably, this included decisions about the form, frequency and length of surveillance imaging and follow‐up consultations. Some decisions were not currently regarded as preference‐sensitive with a low recognition of the need for patient involvement, such as decisions about anti‐hormonal therapy and the management of treatment‐induced menopausal symptoms.

Notably, the data indicated that the patient's role and involvement should be improved for several decisions. First, regarding the form, frequency and length of surveillance imaging, patients desired more frequent (Brandzel et al., 2017; Klaassen et al., 2017) and intensive (Brandzel et al., 2017) surveillance; continuity of care and more frequent or longer appointments were preferences expressed in other studies already (Kimman et al., 2010; Murchie et al., 2016). Despite these strong preferences, patients were rarely involved in making decisions, with physicians typically setting the imaging type and frequency (Brandzel et al., 2017). However, this is probably a legitimate approach because guidelines provide clear, evidence‐based recommendations about surveillance schemes and imaging modalities (Grunfeld et al., 2005; IKNL, 2012; Runowicz et al., 2016; Senkus et al., 2015). We suspect that the identified preferences were primarily based on the patient's need for reassurance (Allen, 2002; Brandzel et al., 2017; Klaassen et al., 2017), and that they may be unaware that more intensive surveillance has no evidence base (Rosselli Del Turco et al., 1994), or that increased exposure might even be harmful (Grunfeld, 2009; Meyer et al., 2019). Efforts should be made to improve patient understanding of the goals of surveillance (Kwast, Drossaert, & Siesling, 2013), specifically at the point of transition from treatment to follow‐up (Brandzel et al., 2017; Schmidt et al., 2016). Furthermore, the frequency and length of surveillance could be determined by recurrence‐risk stratification (Grunfeld, 2009), based on data from nomograms or risk‐calculators. Although Rabin et al. (2013) reviewed 22 cancer prognostic tools, of which 8 focussed on breast cancer, patient involvement with these tools was not discussed. The authors found only limited evidence reporting actual use of these in practice.

Issues also existed for follow‐up consultations aimed at the physical and psychosocial effects of treatment. The available research indicated that patients preferred more frequent consultations than was recommended, that these should be led by specialised oncology providers (Klaassen et al., 2017), and that these should be provided over a longer period of time (Hudson et al., 2012). As literature described unmet needs in information provision about follow‐up, health promotion, late and long‐term effects, or emotional and social needs (Binkley et al., 2012; Chawla et al., 2016; Kent et al., 2012; Meade, McIlfatrick, Groarke, Butler, & Dowling, 2017; Schmidt et al., 2016), these preferences may be the result of these unmet needs. Moreover, 24% of patients sought care from multiple other providers (Hudson et al., 2012), suggesting that referral for personalised care may sometimes be more appropriate than providing general oncology‐led follow‐up. We expect that using patient‐reported outcome measures (PROMs) would help to identify patients’ needs regarding specific forms of care (Black, 2013). PROMs can include symptom‐specific scales about, for instance, physical impairments, sexuality problems, psychosocial problems and body image (Cano, Klassen, Scott, & Pusic, 2013; Wintner et al., 2016). Patients and physicians would be able to discuss the results and subsequently ensure appropriate referrals to physiotherapists, sexologists, gynaecologists, medical social workers, psychologists or plastic surgeons, as necessary.

Decisions about anti‐hormonal treatment had little recognition as preference‐sensitive decisions among physicians, which is somewhat consistent with the 2015 European Society for Medical Oncology guideline. Although this guideline states that follow‐up care should seek to motivate patients to continue anti‐hormonal treatment (Senkus et al., 2015), we should remember that patients must suffer many side effects over a long period of time (Benedict et al., 2017; Bluethmann et al., 2017; Brauer et al., 2016; Cahir et al., 2015; Hershman et al., 2016), and that this often occurs without proper counselling (Benedict et al., 2017; Brauer et al., 2016; Cahir et al., 2015). This leaves patients struggling to cope with difficult symptoms with minimal support (Brauer et al., 2016). Given that therapy adherence depends on perseverance despite side effects (Bluethmann et al., 2017; Brauer et al., 2016), the needs and preferences of patients require more personalised attention in the long term. This may be challenging, particularly for patients confronted with menopausal symptoms, for whom safe and effective evidence‐based options for symptom relief are scarce (Balneaves et al., 2016; Sayakhot et al., 2012). Finally, treatment‐affected fertility in young pre‐menopausal women may conflict with the desire to build a family, producing negative long‐term psychosocial effects (Benedict et al., 2017; Gorman et al., 2011; Hsieh & Huang, 2017). These issues necessitate explicit information provision, counselling and ongoing support to ensure treatment compliance and management of side effects (Cardoso et al., 2012; Howard‐Anderson, Ganz, Bower, & Stanton, 2012; Meade et al., 2017).

### 4.1 Strengths and limitations

Several limitations should be kept in mind when interpreting the results of this study. In the interview and focus‐group studies, the samples included in these studies were small, which may limit the generalisability of the data. However, all the included studies were rated as valuable in the quality assessment.

We considered that the effectiveness of patient involvement or SDM is a separate research topic. Shay and Lafaya concluded that evidence about the association between empirical measures of SDM and patient behavioural and health outcomes is lacking. Given that SDM is not associated with improved outcomes, it should not be considered a goal in itself. However, because outcomes do tend to improve with personalised care, SDM may moderate some other factor (Shay & Lafata, 2015).

### 4.2 Practice implications and recommendations

Currently, there is an international trend towards increased SDM in the diagnosis and treatment of all disease, based on the value‐based healthcare initiative (Porter & Teisberg, 2007). Further personalisation of follow‐up care may lead to care that is not only of greater value for the individual patient, but also to care that is more appropriate from a financial perspective, potentially leading to more responsible use of available healthcare services as well. The process used when deciding on breast reconstruction may be considered an example of best practice for other decisions about follow‐up. Eight studies recommended improvement in information provision (Alderman et al., 2011; Causarano et al., 2015; Fu et al., 2017; Hamnett & Subramanian, 2016; Heller et al., 2008; Morrow et al., 2014; Ogrodnik et al., 2016; Potter et al., 2013), and four reported on decision aids to address these information gaps (Causarano et al., 2015; Heller et al., 2008; Sherman et al., 2016; Temple‐Oberle et al., 2014). Although patient involvement seemed to be more straightforward when making elective decisions about breast reconstruction, true involvement in the decision‐making process requires that patients be given the best available evidence, including details of the risks and benefits (Légaré et al., 2008). When the evidence for a certain decision is low, such as when making decisions about relieving menopausal symptoms, this uncertainty should be outlined by physicians (Politi, Lewis, & Frosch, 2013).

## 5 CONCLUSION

We identified a variety of decisions that can be made about the content or form of follow‐up care for patients with breast cancer. We grouped these into four categories: surveillance for recurrent or secondary breast cancer, consultations for physical and psychosocial (late) effects, recurrence‐risk reduction by anti‐hormonal treatment and improving quality of life. More attention should be given to the patient's role and the involvement in decisions where their input is both relevant and possible. Further personalisation of follow‐up care may lead to care of greater relevance and value to individual patients.

## CONFLICT OF INTEREST

None to declare. The manuscript has been seen and approved by all authors.
