# Supplementary material for: Opportunities for personalised follow‐up care among patients with breast cancer: A scoping review to identify preference‐sensitive decisions
Source: Eur J Cancer Care (Engl). 2019 May 9;28(3):e13092. doi: 10.1111/ecc.13092 (PMC9285605; doi:10.1111/ecc.13092)
Supplement: Supplementary file 1 [file ECC-28-e13092-s001.docx]

**Supplementary Table 3:** selected studies per research question*

| **Author** | **Subject** | **Study design** | | | | | **Answers the following research questions*** | | |
| --- | --- | --- | --- | --- | --- | --- | --- | --- | --- |
|  |  | **Primary study goal** | **Study population (n=, criteria)** | **Study design** | **Outcome measures** | **Most important outcome** | **1** | **2** | **3** |
| Alderman, 2011 | Breast reconstructive surgery | (1) To describe the proportion of mastectomy-treated patients who undergo delayed breast reconstruction; (2) To evaluate the underlying factors that contribute to the decision to receive immediate, delayed, or no breast reconstruction; and (3) To assess the association between receipt of immediate, delayed, or no breast reconstruction with patients’ satisfaction with their surgical decision. | Population-based cohort of mastectomy-treated BC patients who were initially surveyed at time of diagnosis in 2002 and reported to the Los Angeles and Detroit SEER registries. | 5-year follow-up survey | The receipt of immediate and delayed post-mastectomy breast reconstruction, expressed in use of reconstruction, factors associated with reconstruction, decision satisfaction. | Of the 384 mastectomy-treated BC patients in the study, 138 (35.9%) received immediate reconstruction, 44 (11.5%) received delayed reconstruction, and 202 (52.6%) did not receive reconstruction. Factors associated with delayed reconstruction were primarily related to uncertainty about the procedure, concern about cancer surveillance, and low priority. Those without reconstruction demonstrated significant informational needs, which should be addressed with future research efforts. | x | x | x |
| Balneaves, 2016 | Treatment-induced (early) menopause | Identification of BC survivors’ use of complementary therapy (CT) and general information and decision-making needs related to menopausal symptoms. | BC survivors (n=22) and health care professionals (n=5). The sample of women was diagnosed between 2008  and 2010, the majority with ER+ stage II BC. Healthcare practitioners included dietitians, pharmacists,  nurses, general practitioners in oncology, and medical and  radiation oncologists. | Needs assessment by interpretive  descriptive methodology; Focus groups with survivors, interviews with conventional and CT. Thematic, inductive analysis as conducted on the data. | Use of complementary therapies, and general information and decision-making needs related to menopausal symptoms. | Menopausal symptoms have significant negative impact on BC survivors. Close to 70 % of the sample were currently using CTs, including mind-body therapies (45.5 %), natural health products (NHPs) and dietary therapies (31.8 %), and lifestyle interventions (36.4 %). However, BC survivors reported inadequate access to information on the safety and efficacy of CT-options. Survivors also struggled in their efforts to discuss CT with HCPs, who had limited time and information to support women in their CT decisions. Concise and  credible information about CTs was required by BC survivors to support them in making informed and safe decisions about using CTs for menopausal symptom management. | x | x | x |
| Benedict, 2017 | (Long term) adjuvant anti-hormonal therapy | Not clearly specified | Young BC survivors | Not clearly specified | Not clearly specified | Preliminary evidence suggests that prioritizing fertility, along with concerns about side effects, leads to ET noninitiation and early discontinuation. Clinical efforts to improve adherence might need to consider patients’ family-building goals during the course of treatment and to appropriately counsel patients according to their priorities and family-building intentions. Educational materials about family building after cancer are still not consistently available or provided. | x | x | x |
| Bluethmann, 2017 | (Long term) adjuvant anti-hormonal therapy | The aims were to build on survey results to qualitatively explore survivors’ experiences with prescribed AET to (a) describe appraisal and management of AET side effects and (b) deconstruct decisions to initiate, discontinue, or maintain AET. | 452 survivors completed a survey, and 30 took part in telephone interviews. Most interview participants (N = 30) were Caucasian, married, and college educated, with a mean age of 57 years (range = 49–86 years). | Survey, telephone interviews. Mixed-methods explanatory sequence research design with a qualitative emphasis. | Appraisal and management of AET side effects; decisions about initiation, discontinuing, or maintaining AET. | Among adherent survivors, the themes of tolerance of side effects and perseverance were strong. Nonadherent survivors expressed more difficulty managing side effects and perceived fewer benefits when side effects were bothersome. The most common side effects mentioned by all survivors were menopausal symptoms and joint pain; less common side effects were cognitive decline and cardiac distress. Some sought advice from their oncology team. Nonadherent survivors appeared initially motivated to maintain AET but identified a tolerance limit for side effects after which a provider’s recommendation was less influential in their decision to maintain or discontinue AET. | x | x | x |
| Brandzel, 2017 | Form, frequency, and length of follow up | Determining where gaps in care and knowledge could be filled regarding surveillance imaging. | 41 women in California, North Carolina, and New Hampshire (USA). Participants were aged 38–75 years, had experienced stage 0–III BC within the previous 5 years, and had completed initial treatment, but could be still taking adjuvant hormone therapy. Women were selected from the BC Surveillance Consortium (BCSC) registries in the US. | Six focus groups followed by a combination of deductive and inductive thematic analysis | Key themes in experiences and preferences about BC surveillance imaging | Women reported various types and frequencies of surveillance imaging and a range of surveillance imaging experiences and preferences. The most commonly reported pattern of surveillance breast imaging after completing BC treatment was mammography every 3 or 6 months for 1 to 3 years after completion of treatment. Many women experienced discomfort during breast imaging and anxiety related to the examination, primarily because they feared subsequent cancer detection. Women reported trust in their providers and relied on providers for imaging decision-making. However, women wanted more information about the treatment surveillance transition to improve their care. | x | x | x |
| Brauer, 2016 | (Long term) adjuvant anti-hormonal therapy | To understand what factors are associated with persistence and how these medications fit into the broader life context of older BC survivors from the perspectives of the women themselves. | 27 women age 65 years or older who were treated for locoregional BC and had started an AI 4 to 36 months before study enrolment. The women were diagnosed on average at 72 years of age and had diverse racial, ethnic, cultural, marital, and socioeconomic backgrounds. | Grounded theory methodology to conduct in-depth, semi-structured interviews | Decisions about persisting with aromatase inhibitors (AIs) | A total of 27 women were interviewed, and they reported that integrating the AI treatment into daily life posed many challenges. The adverse effects of AIs were difficult to disentangle from what women attributed to comorbid conditions or getting older. This challenge in attribution, coupled with less frequent contact with their oncology team, resulted in many women “winging it” or persisting with the AI despite significant struggles. In particular, participants expressed concerns about the impact of perceived adverse effects on quality of life and ability to carry out social roles. Many reported lack of professional guidance or support with respect to persisting with the AI, especially when adverse effects were present, and relied on a variety of self-management strategies to maintain treatment with the AI. The women often described circumstances, or potential tipping points, under which they might discontinue the AI prematurely. | x | x |  |
| Cahir, 2015 | (Long term) adjuvant anti-hormonal therapy | The aim of this study was to use qualitative methods to investigate influences on adjuvant hormonal therapy Medication-taking behaviour (MTB) in women with stage I–III BC. | Participants were purposively sampled across two cancer centres in Ireland with strata defined by their hormonal therapy MTB. Eligible participants were identified from the cancer centres’ oncology databases and were aged ≥18 years, English speaking, had stage I–III invasive BC at diagnosis and had been prescribed adjuvant hormonal therapy for ≥3 months at the time of study commencement. Thirty-one women participated in interviews (mean age 51 years, SD±10), 14 women were adherent and persistent, 7 women were non-adherent and persistent and 10 women were non-persistent. | This was a qualitative study using semi-structured face-to-face interviews. the Framework  Method with the Theoretical Domains Framework (TDF) informing the analysis framework. | Modifiable influences on hormonal therapy MTB. | Three domains identified both barriers and enablers to hormonal therapy MTB across the three MTB strata: beliefs about consequences, intentions and goals and behaviour regulation, but their influence was different across the strata. Key enablers for adherent/persistent women were identified within the domain beliefs about consequences (BC recurrence), intentions and goals (high-priority), beliefs about capabilities (side effects) and behaviour regulation (managing medication). Barriers were identified within the domain behaviour regulation (no routine), memory, attention and decision processes (forgetting) and environmental context and resources  (stressors) for non-adherent/persistent women and intentions and goals (quality of life), behaviour regulation (temporal  self-regulation), reinforcement, beliefs about consequences (non-necessity) and social influences (clinical support) for non-persistent women. | x | x |  |
| Carter, 2010 | Lifestyle changes | To determine factors associated with selecting between two group physical activity programs. | Participants (n=133) were male and female adult cancer survivors who resided in the Charleston, South Carolina metropolitan area. Inclusion criteria were (a) age 18 years or older, (b) diagnosis of cancer (excluding non-melanoma skin cancer) regardless of time since diagnosis, and (c) sufficient functional status to engage in an exercise program. In our study sample, participants had breast (56%), prostate (8%), female reproductive organ (7%), haematological (6%), colorectal (5%), head and neck (5%), and other cancer types (14%). | The present study is nested in a non-randomized  trial. The parent study was a non-randomized intervention trial to compare the physical and quality-of-life effects of participation  in an 8-week team-oriented dragon boat paddling program  versus a group oriented walking program. | physical activity program chosen and demographic, clinical, physical and psychosocial characteristics. | Roughly equal proportions chose to participate in dragon boat paddling or walking (55% versus 45%). Of the  many variables studied, few were associated with program selection. Compared to those who chose the walking program, those who chose the dragon boat paddling team were more likely to be Caucasians (p=.015) and younger (p=.027), and marginally significantly more like to have cancers other than BC (p=.056) and have greater lower-body strength (p=.062). To meet the needs of  cancer survivors, a menu of physical activity program options may be optimal. | x |  |  |
| Causarano, 2015 | Breast reconstructive surgery | To evaluate the feasibility and effect of a pre-consultation educational group intervention on the decision-making process for breast reconstruction. | Inclusion: adult women ≥18; mastectomy; referred to plastic surgeons for consultation of delayed postmastectomy breast reconstruction. Exclusion: active or atypical BC; not speak English; preferred breast revision or nipple reconstruction only; had a previous consultation with plastic surgeon; cognitive impairment or uncontrolled psychiatric diagnosis. 41 patients were enrolled resulting in a recruitment rate of 72 %. | Pilot study for RCT; Patients randomized to the intervention group participated  in a pre-consultation educational group intervention in  addition to receiving routine education. | Decisional conflict scale, the decision self-efficacy scale, two subscales from the Modified-Perceived Involvement in Care Scale (M-PICS), and one subscale from the BREAST-Q. | The Cohen’s d effect size in reduction of  decisional conflict was moderate to high for the intervention group compared to routine education (0.69, 95 % CI=0.02–1.42), while the effect sizes of increase in decision self-efficacy (0.05, 95 % CI=−0.60–0.71) and satisfaction with information (0.11, 95 % CI=−0.53– 0.76) were small. A higher proportion of patients receiving routine education signed informed consent to undergo breast reconstruction (14/20 or 70 %) compared to the intervention group (8/21 or 38 %) P=0.06. A pre-consultation educational group intervention improves patients’ shared decision-making quality compared to routine preoperative patient education. |  | x | x |
| Corney, 2014 | Fertility management | The aim of this qualitative study was to investigate in detail the fertility-related experiences of young childless women with BC, including the information they received, the fertility preservation options given, and the dilemmas they faced. | Women were eligible for interview provided that they had a first episode of BC six or more months ago, were aged under 45, and therefore considered of child-bearing age, and were currently childless but wanted children in the future. Interviews were conducted with 19 childless women aged below 45 with first episode BC diagnosed at least 6 months before. The women’s ages at diagnosis ranged from 20 to 41 and at interview from 24 to 44. Timing of the interview from diagnosis varied from 6 months to 5 years ago. However, the majority had the diagnosis within the last 3 years. | A qualitative individual interview method; Transcripts were  analysed using the thematic method developed by Braun and Clarke. A simple framework for categorization  of within-case themes was used to order themes from the outset using the topic guide questions. | Themes for fertility decision-making; a set of within-case themes was developed for  each participant. | The amount of information given to women from health professionals varied considerably. Only half were given the opportunity to pursue assisted reproductive techniques prior to chemotherapy. Most women were worried about what the future might hold, including their fertility,  the impact of pregnancy on recurrence, and the health of the child. They were generally given little information or support on these issues. | x |  |  |
| Engelhardt, 2016 | (Long term) adjuvant anti-hormonal therapy | To assess the frequency of use of implicit persuasion during consultations and whether the use of implicit persuasion was associated with expected treatment benefit and/or decision making for decision between hormonal and endocrine therapy. | Stage I&II BC patients treated at oncology outpatient clinics of general teaching hospitals and university medical centres. Eighteen oncologists (56% male; mean age 51 years  [range: 34-66]) included 105 patients. Patients were on average 59 years (range: 35-87), and 53% had stage I disease. | Observational study in consecutive consultations | (1) Unbalanced presentation of benefits and side-effects, (2) presenting treatment recommendations as authorised decisions, (3) creating the illusion of decisional control and (4) persuading patients using (clinical) experience. | A median of five (range: 2e10) implicitly persuasive behaviours were employed per consultation. The number of behaviours used did not differ by disease stage (P Z 0.07), but did differ by treatment option presented (P Z 0.002) and nodal status (P Z 0.01). About 50% of patients with stage I or node-negative disease were steered towards undergoing chemotherapy, whereas 96% of patients were steered towards undergoing endocrine therapy, irrespective of expected treatment benefit. Decisions were less often postponed if more implicit persuasion was used (P Z 0.03). Oncologists frequently use implicit persuasion, steering patients towards the treatment option that they think is in their patients’ best interest. Expected treatment benefit does not always seem to be the driving force behind implicit persuasion. |  | x | x |
| Fasse, 2017 | Breast reconstructive surgery | This study aimed to gain a better understanding of the couples’ decision-making process for BR in the cancer context and particularly to investigate the partners’ involvement in this process. | Eighteen participants (nine women who underwent a mastectomy and their intimate partners) took part in this study. The time of the BC diagnosis varied between 2 and 8 years (M = 5; SD = 2.4). Inclusion criteria were as follows: at least 18 years old, speaking and reading French fluently, being married, and/or living together in a heterosexual long-term relationship at least since the BC diagnosis. For women with DBR, BR had to be finished for at least 6 months. The women of the sample were aged between 33 and 66 years (M = 54, SD = 7.5) and their partner between 40 and 76 years (M = 59, SD = 11.6). | Semi-directive interviews, and a general inductive approach was chosen to capture the  representations of the couples. | Themes and sub-themes in the couples’ decision-making process for BR in the cancer context. During the interviews, the following domains were explored: decision about BR and its potential evolution over time, motivations for decision, individual representations of BR/  no BR, and beliefs and expectations of the surgical procedure. | The analysis revealed 11 major themes. The two most salient ones were ‘external influence’ and ‘implication of the partner’. The exploration of the subthemes  revealed that the decision-making process is often reported as an interrelated experience by the couples and as a dyadic stressor. The partner’s role is depicted as consultative and mostly supportive. | x | x | x |
| Flitcroft, 2016 | Breast reconstructive surgery | To document the reasons women with high-risk BC choose IBR, DBR or no BR (NBR). | Fifty-one women from a metropolitan breast oncology practice, who were likely to require post-mastectomy radiotherapy (PMRT), were recruited after making their decision about BR. The study took place in an oncoplastic  breast surgical practice in metropolitan Sydney, Australia. | Single-site pilot sub-study, questionnaire | Factors that affect the decision about BR classified into eight issue-based domains (feeling normal, feeling good, being practical, influence of others, expectations, fear, timing and unnecessary). | Women over 60 were more likely to choose  NBR (p = 0.005), while women living with a partner were more likely to choose IBR (p = 0.032). The most relevant domains for both IBR and DBR were ‘feeling good’ and ‘feeling normal’; and for NBR were ‘unnecessary’ and ‘being practical’. Although all women understood pre-operatively the potential aesthetic limitations of PMRT, 63% still chose IBR. | x | x | x |
| Fu, 2017 | Breast reconstructive surgery | To investigate cultural factors, values, and perceptions held by Asian women that might impact breast reconstruction rates. | Thirty-five immigrant East Asian women who underwent surgical treatment for BC. The average years lived in America was greater than 27.4, with a range of 3 to 40 years. The mean age of participants was 51 years, with a range from 33 to 72 years. Seventeen patients (48.6%) had undergone at least one mastectomy and reconstruction, 13 patients had undergone at least one mastectomy without reconstruction (37.1%), and five patients had undergone a lumpectomy (14.3%). | Semi-structured interviews with open-ended questions. Each interview session was audio-recorded and transcribed for analysis. Once transcribed, three study investigators trained in qualitative methods independently applied open coding in NVivo software | Recurring themes for cultural factors, values, and perceptions held by Asian women that might impact breast reconstruction rates. | Emerging themes include functionality, age, perceptions of plastic surgery, inconvenience, community/family, fear of implants, language, and information. Patients spoke about breasts as a function of their roles as a wife or mother, eliminating the need for breasts when these roles were fulfilled. Many addressed the fear of multiple operations. Quality and quantity of information, and communication with practitioners, impacted perceptions about treatment. Reconstructive surgery was often viewed as cosmetic. Community and family played a significant role in decision-making. | x | x | x |
| Gorman, 2011 | Fertility management | To gather information about how young women make cancer treatment decisions and to investigate the role of fertility in their decision-making process. | Participants were early stage BC survivors (Stage I or II) diagnosed at age 40 or younger. Women were recruited from the Women’s Healthy Eating and Living (WHEL) study, a multisite randomized, controlled trial to evaluate the effectiveness of a high-vegetable, low-fat diet to reduce recurrence and through a local affiliate of the Young Survival Coalition YSC). Twenty young BC survivors diagnosed with Stage I (30%) or Stage II (70%) BC between the ages of 26 and 38 years participated. Women were recruited from multiple geographic regions and diagnosed between 1 and 13 years prior to the interview. | Telephone interviews, 45 to 75 minutes in duration. We used a semi-structured interview  guide so that each participant was asked a similar set of questions. Questions were open-ended  to facilitate conversation on each topic and participants were encouraged to elaborate on their answers. | Themes and sub-themes for fertility decision-making; cross-case analysis was applied, where data from all participants were combined rather than  analysed as individual cases. | The main themes were: 1) I was young, I wanted to do everything possible to move  forward with my life and not to have the cancer come back, 2) Fertility concerns are different for every woman 3) My oncologist was great… a huge part of my survivorship, and 4) They didn’t tell me about my options and I didn’t think about fertility until it was too late. While fertility was important to many participants, treatment decisions were mainly motivated by survival concerns. Fertility concerns depended on life circumstances and the timing in relation to diagnosis varied. There is a need for improved information regarding the impact of treatment on fertility and fertility preservation options, even if concerns are not expressed at diagnosis. | x | x | x |
| Hamnett, 2016 | Breast reconstructive surgery | The primary aim is to use this evidence to improve the current decision-making process and produce an algorithm that  would guide the patient, the surgeon and the breast care nurse throughout each stage of this decision-making process. | BC patients | Narrative literature review; A literature search was conducted using PubMed, Medline,  evidence.nhs.uk and the Cochrane database. | Factors directing the patient and  reconstructive surgeon | If reconstruction is oncological plausible and co-morbidities and frailty formally assessed, older women should be actively informed about breast reconstruction,  receive support and engage in ’shared decision-making’. The older patient is less likely to do research independently. Amongst other factors, body image, cancer fears, employment and carer responsibilities play a part in the decision. With adequate preoperative and frailty assessment and early involvement of the geriatrician and anaesthetist, microsurgical reconstruction is safe. | x | x | x |
| Heller, 2008 | Breast reconstructive surgery | To assess the effectiveness of an interactive digital education aid for breast reconstruction patients. | BC patients who were candidates for breast reconstruction were recruited and randomized into a control group and a study group. A total of 133 women participated, 66 in the control group and 67 in the study group. | Prospective randomized study | Knowledge, anxiety, and satisfaction before the initial plastic surgery consultation, immediately before surgery, and 1 month after surgery. | An interactive digital education aid is a beneficial educational adjunct for patients contemplating breast reconstruction. Patients who use an interactive digital education aid demonstrate greater factual knowledge, reduced anxiety, and increased postoperative satisfaction compared with patients given preoperative instructions using standard methods alone. |  |  | x |
| Hershman, 2016 | (Long term) adjuvant anti-hormonal therapy | To determine associations of demographic and clinical factors, psychosocial factors, quality of life, and patient treatment satisfaction with the risk of ET non-persistence among women who had initiated it. | Women with BC receiving care in an integrated healthcare system between 2006 and 2010. We identified 601 patients with HR-positive BC who met our initial inclusion criteria. Of these, 523 initiated therapy and had a baseline and subsequent interview. The cohort was primarily white (74.4 %), stage 1 (60.6 %), and on an aromatase inhibitor (68.1 %). | Serial interviews were conducted at baseline and  every 6 months. | The Functional Assessment of Cancer  Therapy (FACT), Medical Outcomes Survey, Treatment  Satisfaction Questionnaire (TSQM), Impact of Events Scale (IES), Interpersonal Processes of Care measure, and  Decision-making beliefs and concerns were measured. | Of the 523 women in our final cohort who initiated ET and had a subsequent evaluation, 94 (18 %) were non-persistent over a 2-year follow-up. At follow-up, the FACT, TSQM, and IES were associated with non-persistence (p\0.001). Most women continued ET. Women who reported a better attitude toward ET, better quality of life, and more treatment satisfaction, were less likely to be non-persistent and those who reported intrusive/avoidant thoughts were more likely to be non-persistent. | x | x |  |
| Holmes, 2017 | Alternative medicine | The objective was to explore BC survivors’ use of the internet when making decisions about complementary and alternative medicine (CAM) use. | 11 BC survivors, from a selection of BC survivors who were 18 years of age or older who had completed active cancer treatment (chemotherapy, radiotherapy and/or surgery) in the last five years, were internet users and had considered using some form of Complementary and Alternative Medicine. | Quantitative questionnaire and a qualitative telephone interview. A mixed-method design was used, combining a qualitative interview study with an embedded questionnaire study. | Participants’ experiences of using the internet to make decisions about CAM and identify the barriers and facilitators to using the internet as a self-management resource. | All participants found information on CAM using the internet and used some form of CAM after their diagnosis. Themes from the interviews went beyond the standard definitions of the TPB areas. Despite the lack of approval from their social network and healthcare team, participants used the internet to find information on CAM. Further, participants’ cancer diagnosis changed their needs, transforming how they perceived and experienced the internet. | x |  | x |
| Hsieh, 2017 | Fertility management | To understand the risk–benefit perception of choosing conception versus contraception among women who have completed BC treatment in Taiwan. | BC survivors who were diagnosed with breast carcinoma before 49 years of age, who finished cancer-related chemotherapy before 50 years of age, who admitted making the decision to conceive after treatment, and who could communicate in Chinese were included in the study. In all, 16 cancer survivors were recruited, with nine women trying to get pregnant and seven women taking contraceptive action. The mean age of all participants at cancer diagnosis was 36.8 years (range, 23–48 years). The average number of years after cancer treatment was 6.8 (range, 1–16 years). | In-depth interviews; grounded theory approach to analyse the  data following the principles of constant comparison. | Risks and benefits of pregnancy after their cancer diagnosis and treatment process, how the decision to conceive or not was reached and whether patients finally tried to conceive | Seven dimensions of risk–benefit perception of pregnancy, including perceived health status, safety, expected gain, harm, loading, support and time were explored among women treated for BC. We found that women treated for BC applied risk–benefit perceptions to decide whether to become pregnant. Implementing contextual counselling could help to decrease perceived barriers to choose pregnancy and increase the quality of pregnancy care. | x | x |  |
| Hudson, 2012 | Form, frequency, and length of follow up | To propose a research, policy, and practice  agenda that advocates for multifaceted decision  support to enhance cancer survivorship and follow-up  care. | 33 cancer survivors, recruited cancer were survivors in New Jersey who had received their cancer treatment  from one of five community hospitals. purposive sample of ambulatory, early stage (I or II) breast and prostate cancer survivors for whom the Institute of Medicine authors recommend longitudinal survivorship health care (i.e.,  defined as ≥2 years from completion of cancer  therapy other than hormonal therapy). Patients with  severe comorbid conditions that require extensive specialist care coordination (e.g., congestive heart failure, myocardial infarction, angina) were excluded. | An exploratory  qualitative study by in-depth, individual interviews. qualitative analysis used a multistep immersion/  crystallization approach. | Survivor experiences | Three types of survivor experiences were identified from narratives of patients treated in community oncology and National Cancer Institute designated comprehensive cancer centers, ranging from nonactivated patients who need enhanced health care communication and decision support to navigate their care to highly activated patients adept at navigating complex health care settings. | x | x | x |
| Kadmon, 2016 | Breast reconstructive surgery | To address decision-making styles among BC survivors considering breast reconstruction. | 70 women who had undergone breast reconstruction surgery in the past five years. The mean age of the participants was 52.7 years (SD = 10.2), with a median age of 52 years. Thirty-eight women had chosen a mastectomy and immediate reconstruction, and the rest had reconstructive surgery later. In 70% of cases, the mastectomy was unilateral; in the rest, it was bilateral. | Single-centre questionnaire | Level of involvement in decision making, decision-making model between provider and patient, and decision-making styles | A statistically significant  correlation was found between the level of involvement in decision making and the decision-making style of the patient. Nurses should assess patient decision-making styles to ensure maximum patient involvement in the decision-making process based on personal desires regardless of age. | x |  | x |
| Klaassen, 2017 | Form, frequency, and length of follow up | The aim of this study was to assess the needs of patients and health professionals with regard to an aftercare decision aid to systematically develop such a decision aid. | 11 female patients who finished their curative BC treatment in one of two medical centres in the southern part of the Netherlands. The average age of the patients was 62 years (range 49–75). | Focus groups and individual interviews. A semi-structured  question guide was used during the focus group  interviews. A similar but adjusted semi-structured question guide was used to conduct the face-to face interviews. | Needs of patients and health professionals with regard to an aftercare decision aid | Although most patients felt few aftercare options were available to them, health professionals reported to provide various options on the patients’ request. Patients reported difficulty in expressing their need for options to their health professional. Although most patients were unfamiliar with decision aids, the majority preferred a paper-based patient decision aid, while most health professionals preferred an online tool. | x |  | x |
| Lee, 2010 | Breast reconstructive surgery | To identify the most important facts and goals for decisions about breast reconstruction after mastectomy, and to compare patients’ and providers’ perspectives. | BC survivors and providers. Survivors (n=21): Those who were older than 21, with a history of early stage BC diagnosed within 5 years prior to contact and treated with mastectomy, and who could speak and read English were eligible. The patient response rate was 79%. Providers (n=20): the provider response rate was 77% in the larger study. | Cross-sectional survey. The study was part of a larger study of  patients’ and providers’ perspectives on reconstruction, surgery (lumpectomy vs. mastectomy), and systemic therapy (chemotherapy  and hormone therapy). | Facts and goals/ concerns related to breast reconstruction after mastectomy. | Providers were more concerned about the impact of radiation on the success of the reconstruction than patients (60% vs. 24%, 95% CI of the difference: 64, 8).  Thirty percent of providers placed the fact that women who do not have  reconstruction are equally satisfied as women who have reconstruction in the  top 3, whereas almost no patients did (30% vs. 5%, 95% CI: 47, 3). For  all 3 of the facts about immediate versus delayed reconstruction, women  placed a higher priority on these facts than providers did. Goals: Patients placed greater importance on avoiding use of a prosthesis (33% vs. 0%, 95% CI of the difference: 13, 54). There was a trend toward less patient concern about “looking natural without clothes” compared to providers (24% vs. 40%, 95% CI of the difference: 12, 44). | x | x | x |
| Morrow, 2014 | Breast reconstructive surgery | To examine correlates of breast reconstruction after mastectomy and to determine if a significant unmet need for reconstruction exists. | Women aged 20 to 79 years diagnosed as having ductal carcinoma in situ or stages I to III invasive BC. The analytic sample for this study consists of the 485 patients who reported undergoing mastectomy at the initial survey, completed the follow-up survey, and indicated that they did not have a recurrence of BC. The mean age was 55.8 years; 42.2% had no more than a high school ducation,and64.3%had stage I or II BC. Black and Latina women were oversampled to ensure  adequate representation of racial/ethnic minorities. | Survey | Breast reconstruction at any time after mastectomy and patient satisfaction with different aspects of the reconstruction decision-making process. | Factors significantly associated with not undergoing reconstruction were black race (adjusted odds ratio [AOR], 2.16 [95%CI, 1.11-4.20]; P = .004), lower educational level (AOR, 4.49 [95%CI, 2.31-8.72]; P < .001), increased age (AOR in 10-year increments, 2.53 [95%CI, 1.77-3.61]; P < .001), major comorbidity (AOR, 2.27 [95% CI, 1.01-5.11]; P = .048), and chemotherapy (AOR, 1.82 [95%CI, 0.99-3.31]; P = .05). Only 13.3% of women were dissatisfied with the reconstruction decision-making process, but  dissatisfaction was higher among non-white patients in the sample (AOR, 2.87 [95%CI,  1.27-6.51]; P = .03). The most common patient-reported reasons for not having  reconstruction were the desire to avoid additional surgery (48.5%) and the belief that it was not important (33.8%), but 36.3%expressed fear of implants. Reasons for avoiding reconstruction and systems barriers to care varied by race; barriers were more common among non-white participants. | x | x | x |
| Neugut, 2012 | (Long term) adjuvant anti-hormonal therapy | To investigate factors related to non-initiation of hormonal therapy among women with newly diagnosed, non-metastatic Hr-positive BC recruited from three U.S. sites. | Women with newly diagnosed, non-metastatic Hr-positive BC recruited from three U.S. sites. Of 1,050 BC patients recruited, 725 (69 %) had HR-positive BC, of whom 87 (12.0 %) based on self-report and 122 (16.8 %) based on medical record/pharmacy fill rates did not initiate hormonal therapy. | A prospective cohort design by interviews | Factors related to non-initiation of hormonal therapy | In a multivariable analysis, non-initiation of hormonal therapy, defined by medical record/pharmacy, was associated with having greater negative beliefs about efficacy of treatment (OR 1.42, 95 % CI 1.18–1.70). Non-initiation was less likely in those who found the quality of patient/physician communication to be higher (OR 0.96, 95 % CI 0.93–0.99), the hormonal therapy treatment decision an easy one to make (OR 0.45, 95 % CI 0.23–0.90) or neither easy nor difficult (OR 0.34, 95 % CI 0.20–0.58); and had more positive beliefs about hormonal therapy efficacy (OR 0.40, 95 % CI 0.34–0.62). | x | x | x |
| Ogrodnik, 2016 | Breast reconstructive surgery | To explore patterns in delayed reconstruction (DR), identify barriers to follow through, and to determine the adequacy of EHR documentation in providing information about decision-making for breast reconstruction. | From the year 2008 to 2012, a total of 367 women were identified undergoing total mastectomy for BC (Fig. 1). One hundred forty-eight women (40.33 %) had IR, either with tissue expanders or autologous tissue. Out of the remaining 219 women, 13 (5.9 %) completed DR. | Retrospective electronic health  record review | The determinants impacting rates of delayed breast reconstruction, potential barriers to follow through with an initial decision to pursue delayed reconstruction, the adequacy of the electronic health record to provide information pertaining to the decision-making process for breast reconstruction. | Of 367 women who had undergone a total mastectomy, 219 did not receive immediate reconstruction. Of these, 24.6 % expressed no interest in DR, 21.9 % expressed interest but were still pending the procedure, and 5.9 % had completed DR. Of decision-making regarding breast reconstruction, 47.5 % lacked documentation. Median follow-up was  34 months. Reasons for not following through with DR included poor timing (25 %), indecision (17 %), desired method  of reconstruction not available at treating facility (10 %), persistent obesity (8.3 %), continued smoking (4 %), and reason  not specified (35 %). Many women do not receive breast reconstruction despite expressing an initial interest in the procedure. Reasons were multi-factorial and the extent of documentation was inconsistent. Further exploration of potential barriers to breast reconstruction as well as opportunities to enhance shared decision-making may serve to improve patient experience and satisfaction following mastectomy. | x | x | x |
| Potter, 2013 | Breast reconstructive surgery | To explore access to  care and the provision of procedure choice to women seeking reconstructive surgery. | Sixty-two interviews were undertaken with 35 health professionals (including two interviews with pairs of CNS) and 31 patients. Interviews were undertaken with OPBS (n=11), plastic surgeons (n=11), CNSs (n=11) and clinical psychologists (n=2) providing specialist reconstructive services at 15 centres throughout the United Kingdom. Thirty-one women with a median age of 51 years (range 31–72 years) who had undergone a range of reconstructive procedures (expander–implant reconstruction n=11; latissimus dorsi (LD) flap reconstruction n=10; DIEP flap reconstruction n=11) were interviewed at a median of 14 months (range 2–37 months) following surgery. Twenty-eight women had undergone reconstruction at the time of mastectomy (IBR) and eight received delayed reconstructive procedures. | Semi-structured interviews; Interviews were transcribed verbatim and  analysed using the constant comparative technique of grounded theory. | Provision of choice in reconstructive surgery, barriers to the provision of choice. | Both patients and professionals expressed concerns about the provision of adequate procedure choice and access to  care. Lack of information and/or time, involvement in decision making and issues relating to the evolution and organisation of  reconstructive services, emerged as potential explanations for the inequalities seen. Interventions to improve cross-speciality collaboration were proposed to address these issues. Inequalities in the provision of choice in BR exist, which may be explained by a lack of integration between surgical specialities. | x | x | x |
| Rini, 2009 | hereditary testing and subsequent risk-behaviour | To investigate high-risk BC survivors’ risk reduction decision making and decisional conflict after an uninformative BRCA1/2 test. | Potential participants were adult, English-speaking women with a history of BC who were probands being tested for BRCA1/2 mutations at three centres between April, 2001 and July, 2004. Women in the sample were, on average, 52 years old (SD = 10 years). Most were married (72%), White (96%), had completed at least some college (96%), and had moderate to high annual household income (median > $75,000). They had been diagnosed with BC nearly six years earlier, on average (M = 5.96, SD = 7.80). Thirty-seven percent had undergone full BRCA1/2 sequencing and the rest had undergone Jewish panel testing. 182 women in the final sample. | Questionnaire, Prospective, longitudinal study of 182 probands undergoing BRCA1/2 testing, with assess-ments 1-, 6-, and 12-months post-disclosure. | Main outcomes included women’s perception of whether they had made a final risk management decision (decision status) and decisional conflict related to this issue. | There were four patterns of decision making, depending on how long it took women to make a final decision and the stability of their decision status across assessments. Late decision makers and non-decision makers reported the highest decisional conflict; however, substantial numbers of women—even early and intermediate decision makers—reported elevated decisional conflict. Analyses predicting decisional conflict 1- and 12-months post-disclosure found that, after accounting for controls and decision status, health beliefs and emotional factors predicted decisional conflict at different timepoints, with health beliefs more important one month after test disclosure and health beliefs more important one year  later. Many of these women may benefit from decision making assistance. | x | x | x |
| Sayakhot, 2012 | Treatment-induced (early) menopause | To investigate the perception and experience of menopause diagnosis and therapies, the information provided and health behaviours in younger women with BC. | The questionnaire study was completed by 114 women, aged 40 – 51 years, with non-metastatic BC. Women were recruited from outpatient clinics and the community. | Questionnaire | Information provision, experience of menopause diagnosis and treatment | Most women were satisfied with the manner in which they were informed of the BC (69%) and the menopause (59%) diagnoses. Although 80% of women were given BC information, only  54% were given menopause information at diagnosis. Women were least satisfied (26%) with information regarding the long-term complications of menopause. Women perceived exercise (68%) and improving lifestyle (61%) as most effective in alleviating symptoms of menopause. The majority of women reported that they did not understand the risks/benefits of ‘ bioidentical ’ hormones (79%) and herbal therapies (78%), while  58% perceived hormone replacement therapies as associated with an increased risk of BC. Most women reported weight gain (68%) and osteoporosis (67%) as the most common problems/fears regarding menopause. However, regarding health behaviours, only 56% reported having relevant tests including a blood sugar test or a bone density test. | x | x | x |
| Sherman, 2016 | Breast reconstructive surgery | To assess the impact of an online decision aid [Breast RECONstruction Decision Aid (BRECONDA)] on breast reconstruction decision-making. | Women (n = 222) diagnosed with BC or ductal carcinoma in situ, and eligible for reconstruction following mastectomy, completed an online baseline questionnaire. They were then assigned randomly to receive either standard online information about breast reconstruction (control) or standard information plus access to BRECONDA (intervention). Forty-five women had undergone bilateral mastectomy for contralateral primary tumours (no women had undergone bilateral prophylactic mastectomy). | Randomized controlled trial | Decisional conflict | Linear mixed-model analyses revealed that 1-month decisional conflict was significantly lower in the intervention group (27.18) compared with the control group (35.5). This difference was also sustained at the 6-month follow-up. Intervention participants reported greater satisfaction with information at 1- and  6-month follow-up, and there was a non-significant trend for lower decisional regret in the intervention group at 6-month follow-up. Intervention participants’ ratings for BRECONDA demonstrated high user acceptability and overall satisfaction. Women who accessed BRECONDA benefited by experiencing significantly less decisional conflict and being more satisfied with information regarding the reconstruction decisional process than women receiving standard care alone. |  |  |  |
| Shtaynberger, 2016 | Lifestyle changes | To validate decisional balance measures for physical activity and fruit and vegetable (F/V) consumption among an adult survivorship population. | Participants were n=86 patients who completed primary treatment for breast or prostate cancer at least 5 years previously and were enrolled in an e-health intervention that aimed to improve physical activity and nutrition behaviours. Participants were pre dominantly non-Hispanic White (81.2%), and female (96.4%), with a mean age of 59.8 (SD=11.4). | Randomized pilot study | Decisional balance, stage of change, fruit/vegetable consumption, and physical activity. | Overall, findings provide validation for these decisional balance measures as indicators of health behaviours and support the value of using these measures in further research to aid understanding of behaviour change in this population. | x | x |  |
| Temple-Oberle, 2014 | Breast reconstructive surgery | To present a heterogeneous group of women treated with the spectrum of breast reconstruction options, and report their satisfaction. | One hundred twenty three of 176 (70%) women completed the questionnaire (43% autologous, 47% alloplastic, and 10% LD/implant reconstructions). The LD/implant group had a low rate of immediate reconstruction (8.3%, P=0.04), and the highest rate of chemotherapy (91.7%, P=0.002) and radiation (100%, P=0.003). | Intervention assessment by questionnaire | Reconstruction satisfaction expressed by ten subscales of the BRECON‐31 | One hundred twenty three of 176 (70%) women completed the questionnaire (43% autologous, 47% alloplastic, and 10% LD/implant reconstructions). The LD/implant group had a low rate of immediate reconstruction (8.3%, P¼0.04), and the highest rate of chemotherapy (91.7%, P¼0.002) and radiation (100%, P¼0.003). The alloplastic group had a high rate of bilateral reconstruction (86.8%, P¼0.01). All groups scored well on the self‐image, arm concerns, intimacy, satisfaction, and expectations subscales. All groups scored moderately on the self‐consciousness, appearance, and nipple subscales. The autologous group scored the lowest on recovery (51 vs. 68 and 65, P<0.0001) and only moderately well on the abdomen subscale (67). Multiple regression analysis showed that satisfaction was not driven by type of reconstruction (P>0.05). |  |  |  |
| Wandrey, 2015 | Breast reconstructive surgery | To explore lesbian BC survivors' attitudes toward breast reconstruction. This study represents the first published study to analyse data from a lesbian-specific BC forum to evaluate such attitudes. | Lesbian BC survivors; Two hundred fifty-five users posted to the lesbian-specific forum; 53 of these users discussed breast reconstruction and were included in the present analysis. We analysed a total of 168 posts. | Online support forum analysis | Lesbian BC survivors’ attitudes toward breast reconstruction. | Our analysis revealed five important themes related to breast reconstruction attitudes as follows: (1) rejecting being defined by their body image, (2) privileging sensation over appearance, (3) believing that being breastless is protective, (4) perceiving their social context as supportive of non-reconstruction, and (5) feeling pressured by social norms to undergo reconstructive surgery. | x | x |  |
| Zielinski, 2015 | Breast reconstructive surgery | To investigate the reasons why women after amputation of the breast due to cancer are not likely to undergo breast reconstructive surgery. | 73 women from Silesian province aged 37-79 who undergone breast amputation due to malignant neoplasm in 1987-2013. The mean age of examined women was 58 years. The largest group were patients in their sixties (n=34). | Questionnaire | Reasons given by women for refraining from breast reconstruction | From all of the reasons given by women for refraining from breast reconstruction, the most frequently pointed was the fear of being subjected to further surgery (38.3%). 23 women (31.5%) admitted that they were also afraid of postoperative pain. Similarly, a common response (35.6%) was that  it is not essential for their mental state, and 30% of respondents fully accepted their appearance after mastectomy. Concern about the effect of failed reconstruction was reported by 24.6% of the women, and the fear that the surgery could negatively affect the process of cancer treatment by 27.4% of respondents. Lack of information about the capabilities and knowledge of breast reconstruction methods was not an important factor in decision-making. Most of the surveyed women who abandon breast reconstruction surgery, make this decision on the basis of more than one reasons. | x | x | x |
| * 1) What are the common complaints and issues that can occur for woman treated for BC with curative intent for which decisions have to be made with regard to management within five years after curative treatment?; 2) to what extent are decisions with regard to the management of these complaints preference-sensitive?; 3) To what extent and how are BC patients involved in making these follow-up-related decisions? | | | | | | | | | |
