# Supplementary material for: Opportunities for personalised follow‐up care among patients with breast cancer: A scoping review to identify preference‐sensitive decisions
Source: Eur J Cancer Care (Engl). 2019 May 9;28(3):e13092. doi: 10.1111/ecc.13092 (PMC9285605; doi:10.1111/ecc.13092)
Supplement: Supplementary file 2 [file ECC-28-e13092-s002.docx]

**Supplementary Table 2:** Critical Appraisal Skills Programme (CASP) quality scoring according to the study design performed, for all included studies

| **Author** | **Study design** | **Quality scoring criteria, according to the study design*** | | | | | | | | | | | | |
| --- | --- | --- | --- | --- | --- | --- | --- | --- | --- | --- | --- | --- | --- | --- |
|  |  | **1** | **2** | **3** | **4** | **5** | **6** | | **7** | **8** | **9** | **10** | **11** | **12** |
| Alderman, 2011 [[42](#_ENREF_42)] | Cohort | Y | Y | Y | Y | Y | Y | | Woman who received IBR did differ from those who received DBR or NBR by several clinical factors | ? | Y | Y | Y | Education, improved information provision and decision-making, and develop and deploy decision tools |
| Balneaves, 2016 [[36](#_ENREF_36)] | Qualitative | Y | Y | Y | Y | Y | ? | | ? | Y | Y | V | n/a | n/a |
| Benedict, 2017 [[29](#_ENREF_29)] | Qualitative | N | ? | ? | ? | Y | ? | | ? | N | Y | V | n/a | n/a |
| Bluethmann, 2017 [[30](#_ENREF_30)] | Qualitative, Quantitative | Y | Y | Y | Y | Y | ? | | Y | N | Y | V | n/a | n/a |
| Brandzel, 2017 [[26](#_ENREF_26)] | Qualitative | Y | Y | Y | Y | Y | ? | | Y | N | Y | V | n/a | n/a |
| Brauer, 2016 [[31](#_ENREF_31)] | Qualitative | Y | Y | Y | N | Y | ? | | Y | ? | Y | V | n/a | n/a |
| Cahir, 2015 [[32](#_ENREF_32)] | Qualitative | Y | Y | Y | Y | Y | ? | | Y | Y | Y | V | n/a | n/a |
| Carter, 2010 [[56](#_ENREF_56)] | RCT | Y | N | Y | N | Y | Y | | The combined average attendance was 74%, and on average paddlers had significantly better attendance than walkers (p = 0.0059) | ? | ? | Y | Y | n/a |
| Causarano, 2015 [[38](#_ENREF_38)] | RCT | Y | Y | Y | N | Y | Y | | The decrease in decisional conflict was greater In the intervention group compared to routine education (*d* 0.69) | 95% CI = -0.60 to 0.71 | Y | Y | Y | n/a |
| Corney, 2014 [[53](#_ENREF_53)] | Qualitative | Y | Y | Y | Y | Y | ? | | ? | Y | Y | V | n/a | n/a |
| Engelhardt, 2016 [[33](#_ENREF_33)] | Qualitative | Y | Y | Y | Y | Y | ? | | Y | Y | Y | V | n/a | n/a |
| Fasse, 2017 [[46](#_ENREF_46)] | Qualitative | Y | Y | Y | Y | Y | ? | | Y | Y | Y | V | n/a | n/a |
| Flitcroft, 2016 [[39](#_ENREF_39)] | Pilot, Cohort | Y | Y | Y | Y | Y | Y | | The range of reasons why woman chose IBR, DBR, and NBR. Utility of domains | ? | Y | ? | Y | BR decision-making and to discuss all options fully with women |
| Fu, 2017 [[47](#_ENREF_47)] | Qualitative | Y | Y | Y | Y | Y | ? | | Y | N | Y | V | n/a | n/a |
| Gorman, 2011 [[54](#_ENREF_54)] | Qualitative | Y | Y | Y | Y | Y | Y | | yes | N | Y | V | n/a | n/a |
| Hamnett, 2016 [[48](#_ENREF_48)] | Review | Y | Y | ? | N | n/a | Evidence-based recommendations for decision-making about BR in older women | | ? | Y | Y | Y | n/a | n/a |
| Heller, 2008 [[50](#_ENREF_50)] | RCT | Y | Y | Y | N | Y | Y | | Interactive digital education aid group showed a sign greater improvement in their knowledge level (p = 0.02) and were more satisfied (p = 0.03) | ? | Y | Y | Y | n/a |
| Hershman, 2016 [[34](#_ENREF_34)] | Qualitative | Y | Y | Y | Y | Y | ? | | Y | Y | Y | V | n/a | n/a |
| Holmes, 2017 [[64](#_ENREF_64)] | Qualitative, Quantitative | Y | Y | Y | Y | Y | Y | | Y | N | Y | V | n/a | n/a |
| Hseish, 2017 [[55](#_ENREF_55)] | Qualitative | Y | Y | Y | Y | Y | ? | | Y | Y | Y | V | n/a | n/a |
| Hudson, 2012 [[28](#_ENREF_28)] | Qualitative | Y | Y | Y | Y | Y | ? | | Y | Y | Y | V | n/a | n/a |
| Kadmon, 2016 [[51](#_ENREF_51)] | Cohort | Y | Y | Y | Y | Y | Y | | Patient age did not correlate with decision-making style or declared level of involvement in the decision-making process for BR | ? | Y | N | Y | Understanding the decision-making process about BR and increasing knowledge on the subject |
| Klaassen 2017 [[25](#_ENREF_25)] | Qualitative | Y | Y | Y | Y | Y | Y | | Y | Y | Y | V | n/a | n/a |
| Lee, 2010 [[44](#_ENREF_44)] | Cohort | Y | Y | Y | Y | Y | Y | | Substantial variability existed among patients and between patients and providers about the key facts for BR decisions | ?  (various 95% CIs) | Y | N | Y | Attention to the most important goals and concerns of women |
| Morrow, 2014 [[52](#_ENREF_52)] | Qualitative | Y | Y | Y | Y | Y | Y | | Y | Y | Y | V | n/a | n/a |
| Neugut, 2012 [[35](#_ENREF_35)] | Cohort | Y | Y | Y | Y | ? | ? | | Factors influencing non-initiation of adjuvant hormonal therapy are complex and influenced by patient belief about treatment efficacy and side effects | Precise (relatively small 95% CI) | Y | ? | Y | Educational interventions may be used in the future to improve initiation and adherence |
| Ogrodnik, 2016 [[45](#_ENREF_45)] | Cohort | Y | Y | Y | Y | Y | Y | | Many women do not receive BR despite interest, but the reasons are both patient- and provider-related | ? | Y | N | Y | Clinicians need to be more explicit about patient preference at every decision |
| Potter, 2013 [[40](#_ENREF_40)] | Qualitative | Y | Y | Y | Y | Y | Y | | Y | ? | Y | V | n/a | n/a |
| Rini, 2009 [[27](#_ENREF_27)] | Qualitative | Y | Y | Y | Y | Y | Y | | Y | N | Y | V | n/a | n/a |
| Sayakhot, 2012 [[37](#_ENREF_37)] | Qualitative | Y | Y | Y | Y | Y | Y | | Y | N | Y | V | n/a | n/a |
| Sherman, 2016 [[43](#_ENREF_43)] | RCT | Y | Y | Y | N | Y | Y | | Use of the decision aid reduced conflict about decisions and improved satisfaction with information about BR choice | Precise | Y | Y | Y | n/a |
| Shtaynberger, 2016 [[57](#_ENREF_57)] | RCT | Y | Y | Y | N | N | Y | | The estimated effect sizes were 1.94 (pros) and −1.43 (cons) | Precise | N | Y | Y | n/a |
| Temple-Oberle, 2014 [[49](#_ENREF_49)] | Cohort | Y | Y | Y | Y | Y | ? | | Various  (8.3% to 100%) | Precise | Y | Y | Y | n/a |
| Wandrey, 2015 [[65](#_ENREF_65)] | Qualitative | Y | Y | Y | N | Y | ? | | Y | N | Y | V | n/a | n/a |
| Zielinski, 2015 [[41](#_ENREF_41)] | Qualitative | Y | Y | Y | Y | Y | ? | | ? | N | Y | V | n/a | n/a |
| **Key:**? = unable to tell; N = no; n/a = not applicable; P, Precise; V = Valuable; Y = yes.  **Abbreviations:**  BR: breast reconstruction  CI: confidence interval  IBR: immediate breast reconstruction  NBR: no breast reconstruction  RCT: randomized controlled trial  *** Critical Appraisal Skills Programme criteria per study type:** | | | | | | | | | | | | | | |
| **Qualitative study**   1. Was there a clear statement of the aims of the research? 2. Is a qualitative/ quantitative methodology appropriate? 3. Was the research design appropriate to address the aims of the search? 4. Was the recruitment strategy appropriate to the aims of the research? 5. Was the data collected in a way that addressed the research issue? 6. Was the relationship between researcher and participants adequately considered? 7. Have ethical issues been taken into consideration? 8. Was the data analysis sufficiently rigorous? 9. Is there a clear statement of findings? 10. How valuable is the research? | | | | | | | | **RCT**   1. Did the trial address a clearly focused issue? 2. Was the assignment of patients to treatments randomised? 3. Were all of the patients who entered the trial properly accounted for at its conclusion? 4. Were patients, health workers and study personnel ‘blind’ to treatment? 5. Were the groups similar at the start of the trial? 6. Aside from the experimental intervention, were the groups treated equally? 7. How large was the treatment effect? 8. How precise was the estimate of the treatment effect? 9. Can the results be applied to the local population, or in your context? 10. Were all clinically important outcomes considered? 11. Are the benefits worth the harms and costs? | | | | | | |
| **Cohort**   1. Did the study address a clearly focused issue? 2. Was the cohort recruited in an acceptable way? 3. Was the exposure accurately measured to minimise bias? 4. Was the outcome accurately measured to minimise bias? 5. A. Have the authors identified all important confounding factors?  B. Have they taken account of the confounding factors in the design and/or analysis? 6. A. Was the follow-up of subjects complete enough?  B. Was the follow-up of subjects long enough? 7. What are the results of this study? 8. How precise are the results? 9. Do you believe the results? 10. Can the results be applied to the local population? 11. Do the results of this study fit with other available evidence? 12. What are the implications of this study for practice? | | | | | | | | **Systematic** **review**   1. Did the review address a clearly focused question? 2. Did the authors look for the right type of papers? 3. Do you think all the important, relevant studies were included? 4. Did the review’s authors do enough to assess quality of the included studies? 5. If the results of the review have been combined, was it reasonable to do so? 6. What are the overall results of the review? 7. How precise are the results? 8. Can the results be applied to the local population? 9. Were all important outcomes considered? 10. Are the benefits worth the harms and costs? | | | | | | |
